# An Ecological Assessment of the Pandemic Threat of Zika Virus

**DOI:** 10.1371/journal.pntd.0004968

**Published:** 2016-08-26

**Authors:** Colin J. Carlson, Eric R. Dougherty, Wayne Getz

**Affiliations:** 1 Department of Environmental Science, Policy, and Management, University of California, Berkeley, Berkeley, California, United States of America; 2 School of Mathematical Sciences, University of KwaZulu-Natal, Durban, South Africa; Centers for Disease Control and Prevention, UNITED STATES

## Abstract

The current outbreak of Zika virus poses a severe threat to human health. While the range of the virus has been cataloged growing slowly over the last 50 years, the recent explosive expansion in the Americas indicates that the full potential distribution of Zika remains uncertain. Moreover, many studies rely on its similarity to dengue fever, a phylogenetically closely related disease of unknown ecological comparability. Here we compile a comprehensive spatially-explicit occurrence dataset from Zika viral surveillance and serological surveys based in its native range, and construct ecological niche models to test basic hypotheses about its spread and potential establishment. The hypothesis that the outbreak of cases in Mexico and North America are anomalous and outside the native ecological niche of the disease, and may be linked to either genetic shifts between strains, or El Nino or similar climatic events, remains plausible at this time. Comparison of the Zika niche against the known distribution of dengue fever suggests that Zika is more constrained by the seasonality of precipitation and diurnal temperature fluctuations, likely confining autochthonous non-sexual transmission to the tropics without significant evolutionary change. Projecting the range of the diseases in conjunction with three major vector species (*Aedes africanus*, *Ae*. *aegypti*, and *Ae*. *albopictus*) that transmit the pathogens, under climate change, suggests that Zika has potential for northward expansion; but, based on current knowledge, our models indicate Zika is unlikely to fill the full range its vectors occupy, and public fear of a vector-borne Zika epidemic in the mainland United States is potentially informed by biased or limited scientific knowledge. With recent sexual transmission of the virus globally, we caution that our results only apply to the vector-borne transmission route of the pathogen, and while the threat of a mosquito-carried Zika pandemic may be overstated in the media, other transmission modes of the virus may emerge and facilitate naturalization worldwide.

## Author Summary

A combination of media attention and the declaration of a World Health Organization state of emergency have made the pandemic expansion of Zika virus a topic of great public concern. Understanding the threat North America faces from the still-expanding viral range requires an understanding of the historical range and ecology of the disease, a topic currently difficult to study due to incomplete occurrence data. We compile the most comprehensive geospatial dataset of Zika occurrences in its native range, beginning with its discovery in 1947, and build bioclimatic models that set an outer bound on where the virus is likely to persist. Our results suggest Zika is likely far more constrained than the closely-related dengue fever, on which many projections have been based. While Zika poses a serious threat in current outbreak regions and is clearly a high-priority neglected tropical disease, our models suggest that even under an extreme climate change scenario for 2050, the disease is unlikely to become cosmopolitan in most temperate regions as a vector-borne disease, a discrepant finding from the results of non-ensemble modeling methods. Despite that, sexual transmission remains a serious public health concern, and a route by which Zika could become a severe public health emergency in temperate zones, including in the United States.

## Introduction

Following a twenty-fold upsurge in microcephalic newborns in Brazil linked to Zika virus (ZIKV), the World Health Organization has declared an international health emergency. [**1**] Despite being profiled for the first time in 1947. [**2**] Zika remained poorly characterized at a global scale until the last six months. Thus, the present pandemic expansion in the Americas poses a threat of currently unknown magnitude. Closely related to dengue fever, Zika conventionally presents as a mild infection, with 80% of cases estimated to be asymptomatic. [**3**] The cryptic nature of infection has resulted in sporadic documentation of the disease and rarely includes spatially explicit information beyond the regional scale. [**1**, **4–6**] This greatly limits the confidence with which statistical inferences can be made about the expansion of the virus. With an estimated 440,000–1,300,000 cases in Brazil in 2015, [**3**] and continuing emergence of new cases in Central America and, most recently, the United States, assessing the full pandemic potential of the virus is an urgent task with major ramifications for global health policy.

Current evidence portrays the global spread of ZIKV as a basic diffusion process facilitated by human and mosquito movement, a hypothesis supported by the frequency of infected traveler case studies in the Zika literature. [**7–10**] Tracing phylogenetic and epidemiological data has revealed the expansion of ZIKV has occurred in a stepwise process through the South Pacific, moving the disease from Southeast Asia into French Polynesia and the Philippines, and subsequently to Easter Island. [**1**, **4–6**] Based on phylogenetic reconstruction, ZIKV is assumed to have dispersed into South America as recently as three years ago from the last of those locations, [**11**] and the virus is presumed to be at a biogeographic disequilibrium in the Americas. With cases in the ongoing outbreak in Colombia, El Salvador, Guatemala, Paraguay, and Venezuela, and by November of last year, as far north as Mexico and Puerto Rico, the full potential distribution of the disease remains unknown. Moreover, several alternative explanations for the disease’s expansion remain overlooked; most notably, the role of climate change in Zika’s expansion has not yet been thoroughly investigated. [**12**]

We present three competing hypotheses that describe the path of expansion that Zika could take, based on evaluations of the ecological niche of the virus within and outside of its vectors. First, if Zika has no additional climatic constraints relative to those of its vectors, future range expansions should match mosquito ranges. Second, if Zika has a transmission niche that is constrained by climatic factors within the ranges of its mosquito vectors, its range may be much more limited—with, as we show below, possible confinement to the tropics—and cases in North America could be driven by human dispersal or extreme episodic weather events. Finally, it is possible that the expansion of Zika into North America may be a steady range expansion beyond the known niche in its native range, facilitated by climatic shifts or by genetic shifts in the virus or vectors. To test these hypotheses, we present a spatially explicit database of Zika occurrences from the literature and an ensemble of ecological niche models [**13**] using that data to map the potential distribution of the virus.

## Methods

### Occurrence Data

Occurrence data for Zika virus was compiled from the literature from studies dating as far back as the original discovery of the virus in Zika Forest, Uganda in 1947. While the asymptomatic nature of the virus limits the total availability of data, lack of evidence for spatial patterns in symptoms in the native range suggest this is an unlikely cause of spatial bias (and instead, merely limits total dataset size). Special attention was paid to correctly attributing cases of travelers to the true source of infection. Locality data was extracted from a combination of clinical cases and seropositivity surveys in humans and mosquitoes, and georeferenced using a combination of Google Maps for hospitals and the Tulane University GEOLocate web platform for the remainder, [**14**] which allows for the attribution of an uncertainty radius to points only identified to a regional level. Four points were georeferenced in the New World but excluded from niche models because a limited sample as small as four points was likely to significantly bias predictions (compared to the necessary number of pseudoabsences in the same region). Thus, sixty points from the Old World were used in the final models presented in our paper after eliminating data from the current outbreak in the Americas. All points included in our dataset had an outer-bound of at most 65 km of uncertainty, with most substantially less. Constraining datasets based on an uncertainty threshold will become more statistically feasible in future studies once more survey data become available. In the present study, we deemed that the additional information gained from each point outweighed the potential impact of the uncertainty on model performance (S1 Table). We note that for similar reasons, we did not subsample our dataset for spatial thinning in our main models, as software packages like spThin allow, [**15**] due to information-accuracy tradeoffs; and the strong final performance of models (and the correspondence of our predictions for dengue and *Aedes* species to published “gold standard” niche models) speaks to the appropriateness of the underlying data and variables. Sensitivity analyses in the literature unequivocally suggest that accuracy of the modeling methods we employ plateaus at or near 50 points, justifying the use of a dataset of this size. [**16–18**]

Occurrence data for the other species included in our study were compiled from the literature. For *Aedes africanus*, we used a dataset of 99 points downloaded from the Global Biodiversity Informatics Facility (www.gbif.org). GBIF’s coverage of *Aedes aegypti* and *Aedes albopictus* was deemed to be lacking, so occurrences for those species were taken from the previously published work of Kraemer *et al*. [**19–20**] Finally, Messina *et al*.’s database was used for dengue, [**21**] as it has been previously published and used with great success to generate a global distribution model. [**22**] Both of these datasets were reduced down to point-only data (i.e., polygons of occurrence were excluded), leaving 5,216 points for dengue and 13,992 and 17,280 points for *Ae*. *aegypti* and *Ae*. *albopictus* respectively.

A number of other Zika vectors are known from previous reports, including at least a dozen *Aedes* species, as well as *Anopheles coustani*, *Culex perfuscus*, and *Mansonia uniformis*. [**23–24**] While we do not include these vectors in this study in order to keep focus on the most likely globally-cosmopolitan *Aedes* vectors, we note these species could be important in regional patterns of establishment. These species lack the globally comprehensive datasets that dominant arbovirus-vectoring *Aedes* species have, and require future attention by similarly-dedicated researchers.

### Ecological Niche Modeling

Due to the potentially transient nature of the New World distribution of Zika virus, our model uses presence and 1000 randomly selected pseudo-absence points from the Eurasian, African, and Australian regions where the virus is established. We used the WorldClim data set BIOCLIM at 2.5 arcminute resolution, an aggregated dataset across values from 1950 to 2000, to provide all but one of our climate variables. [**25**] The BIOCLIM features 19 variables (BIO1-BIO19) that summarize trends and extremes in temperature and precipitation at a global scale. Given the relevance of the normalized difference vegetation index (NDVI) in previous studies of dengue and as a predictor of vector mosquito distributions, [**26**] we downloaded monthly average NDVI layers for each month in 2014 from the NASA Earth Observations TERRA/MODIS data portal, [**27**] at a resolution of 0.25 degrees to maintain compatibility with the BIOCLIM layers (0.25 degrees is equivalent to 15 arcminutes). The twelve monthly layers were averaged to provide a single mean NDVI layer. Due to the absence of NDVI data at the necessary resolution associated with many of the historical records (especially prior to 1992), the use of a recent mean NDVI layer was deemed the most pragmatic method of including vegetation in our models. We also make the simplifying assumption that areas of prior presence correspond to areas of current presence, an assumption that allows the use of current NDVI and is relatively standard for the niche modeling literature.

Species distribution models were executed using the BIOMOD2 package in R 3.1.1, which produces ensemble species distribution models using ten different methods: general linear models (GLM), general boosted models or boosted regression trees (GBM), general additive models (GAM), classification tree analysis (CTA), artificial neural networks (ANN), surface range envelope (SRE), flexible discriminant analysis (FDA), multiple adaptive regression splines (MARS), random forests (RF), and maximum entropy (MAXENT). [**28**] The BIOMOD algorithm runs a series of distribution models using training data, each of which is subsequently weighted and stacked across methods based on relative predictive performance with test data. As Thuiller *et al*. note, if a single modeling method is consistently most accurate, use of that method should be favored over ensemble approaches, [**28**] but in our study model performance varied, making ensemble approaches informed by degree-of-belief in a given model the most powerful option available. With recent publication of two Zika niche modeling papers using MAXENT and boosted regression trees, respectively, [**29–30**] differences between these two modeling methods may be responsible for differences in predictions–an issue that makes ensemble models particularly robust to idiosyncrasies of any individual methods.

Models were run individually for Zika (ZIKV), dengue (DENV), *Ae*. *aegypti*, *Ae*. *albopictus*, and *Ae*. *africanus*. For Zika, models trained on Old World environmental data (from Europe, Africa, Asia and Australia) were used to establish the potential distribution of the virus in the Americas under climatic conditions captured by WorldClim data, which are an aggregate of data between 1950 and 2000 (appropriately matching the date range of historical Zika occurrence data), and represent an expected range of variability that does not incorporate anomalous events like 2015 El Niño Southern Oscillation. Extrapolation between continents is a procedure with the potential for error: if novel environments exist in the New World with incomparable covariance structure between climate variables, predictive accuracy is likely to decline. While using only Old World data could potentially bias our models towards a subset of the niche, this can be readily tested for, by comparing models that include or exclude South American occurrence data.

To address colinearity in the environmental variable set, we produced a correlation matrix for our 20 variables, and identified each pair with a correlation coefficient > 0.8. For each species, we ran a single ensemble model with all ten methods and averaged the variable importance for our 20 predictors across the methods (S2–S6 Tables). In each pair we identified the variable with the greater contribution, and we produced species-specific reduced variable sets used in the final published models by eliminating any covariates that universally performed more poorly than their pair-mate. Based on this criterion, we excluded the following variables for each species to reduce colinearity:

- ZIKV: BIO8, BIO9, BIO14, BIO18
- DENV: BIO3, BIO5, BIO12, BIO17
- *Ae*. *aegypti*: BIO6, BIO8, BIO12, BIO17
- *Ae*. *africanus*: BIO5, BIO6, BIO12, BIO17
- *Ae*. *albopictus*: BIO8, BIO9, BIO16, BIO17

The AUC of every model run with reduced variable sets is presented in S7 Table. We found no significant correlation between NDVI and any individual BIOCLIM variable, so NDVI was included in every model of current distributions. We ran five iterations of each reduced variable set model and eliminated any prediction methods from the ensemble with an AUC of lower than 0.95, so that the final model had only included the best predicting models. This greatly limited the models available for ZIKV and DENV, so a cutoff of 0.9 was applied in those cases, to keep the ensemble approach constant across datasets. The final models were run with the following methods with ten iterations using an 80/20 training-test split in the final presentation:

- ZIKV: GLM, GBM, GAM, CTA, FDA, MARS, RF
- DENV: GLM, GBM, GAM, FDA, MARS, RF, MAXENT
- *Ae*. *aegypti*: GLM, GBM, GAM, CTA, ANN, FDA, MARS, RF
- *Ae*. *africanus*: GLM, GBM, GAM, CTA, ANN, FDA, MARS, RF
- *Ae*. *albopictus*: GLM, GBM, GAM, CTA, FDA, MARS, MAXENT, RF

The importance of variables of the reduced model set for each are presented in S8–S13 Tables, and the final ensemble models are projected from the BIOMOD output in S1–S5 Figs.

### Model Validation

To assess the transferability of our Zika model across environmental space, we conducted a geographic cross validation (GCV) between African and Asian datasets (an analysis we did not repeat for *Aedes* species or dengue, given the far greater sample size and geographic coverage of those species, and the publication of more intensive niche modeling efforts by experts for those systems). While under normal circumstances, a model would be trained on New World data and projected onto the Old World to cross-validate results, the lack of data prior to the current outbreak makes such a direct comparison infeasible. However, given the evidence for separate Asian and African strains, a cross-validation between the two was supported, and models trained on those two continents were projected globally to test the performance of the model across geographic regions, and evaluate how sensitive our projections in the Americas are to the environmental covariates sampled. The clustering of points in western India narrows the environmental range sampled by presences, potentially limiting the apparent transferability of the Asian sub-model. In contrast, the African sub-model performs well in new regions, and corresponds well to the global model.

### Climate Change Projections

The potential contribution of climate change to Zika’s current expansion, and the outer bounds of transmission under future expansion, are largely unaddressed. While these have not been the subject of any concerted speculation, Shapshak *et al*. [**31**] point out that the majority of arboviruses are potentially implicated in the climate change-driven expansion of global disease burden, with a shared set of drivers that quite probably extends to Zika as well. Consequently this analysis serves two purposes; to address the potential expansion and thereby assist public health planning, and to test whether even a liberal post-climate-change interpretation of range margins matches the predictions of Messina *et al*. [**29**] and Samy *et al*. [**30**] that we consider limited in specificity and potentially over-predictive. To project the distribution of the species under a worst-case scenario for climate change, we reran each model with the previously chosen method and variable sets but excluded NDVI, as future values could not be simulated effectively. BIOCLIM forecasts were taken from WorldClim using the Hadley Centre Global Environmental Model v. 2 Earth System climate forecast (HadGEM2-ES) predictions for representative climate pathway 8.5 (RCP85), which, within that model, represents a worst-case scenario for carbon emissions and climate warming. [**32**] All five species’ models were retrained on current climate data and projected onto forecasts for the year 2050. While we could have also included milder climate change forecasts and scenarios in our analysis, public concern over the future spread of Zika make the worst case scenario the most relevant question of interest for public health research (and intermediate scenarios would fall between current ranges and the worst case scenario we project).

### Niche Comparison

To compare the niche of dengue and Zika and thereby address whether dengue models can be appropriately used to forecast the Zika pandemic, we used the R package ecospat, which uses principal component analysis to define the position of species’ ecological niche relative to background environmental variation. [**33–34**] The ecospat analysis was run using the full 64 point database and the full extent of global environmental data, because, while the niche of Zika in the Americas is uncertain, dengue is well established, and the analysis was most appropriately done with global coverage. Niche similarity tests were run with 500 iterations and using the entire set of 20 environmental variables (BIOCLIM + NDVI).

### Model Comparison with Global Data Coverage

Our study is centered on the assumption that incorrect predictions at the country level can have drastic consequences for the misinterpretation of science. As a final precautionary analysis, we supplemented the data published in the Messina *et al*. study [**30**] to our own for a final re-analysis. Broennimann & Guisan [**35**] recommend the pooling of data from native and invasive ranges for ecological niche modeling during the course of a biological invasion, an approach we adopt in this final analysis. The Messina data is heavily clustered in Brazil, with a high degree of aggregation, and especially compared against our less-aggregated, smaller dataset this made the combination of datasets potentially inaccurate. To address this problem, the 390 pooled points were reduced down to 242 points using the package spThin, [**15**] with a 40km buffer between points (the width of an average grid cell for our environmental data). Models were rerun using the same variable and model set as for the primary Zika model and the results of the analysis are included in the supplementary information as S6 Fig and, with a threshold applied based on the true skill statistic, S7 Fig. The final model performs poorer than our main ensemble (weighted model: AUC = 0.970), and while it more appropriately predicts presences in southern Brazil, it does a far poorer job in the rest of the world, once again most likely due to the relative balance of points even after thinning the dataset.

## Results

**Fig 1:**
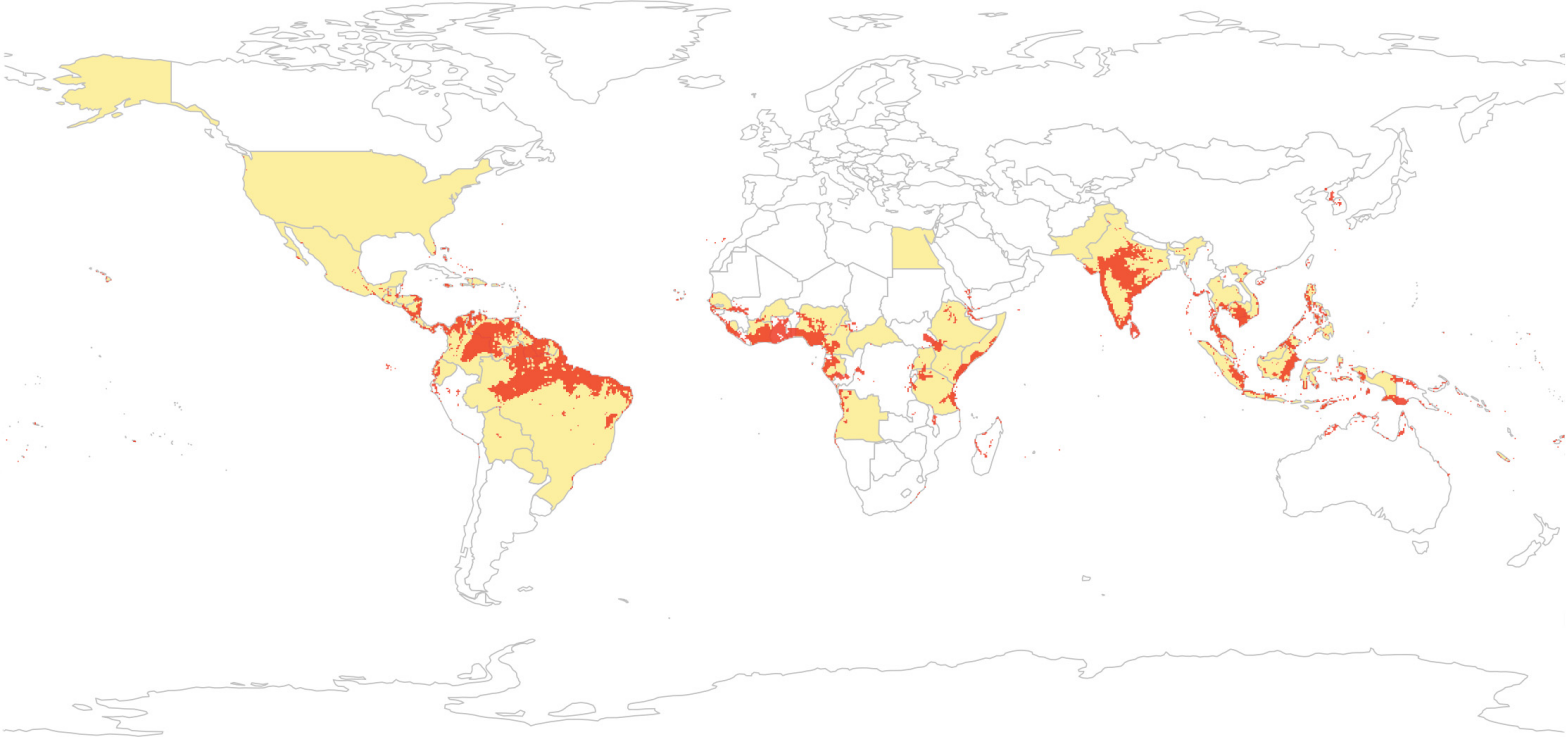
The global distribution of case reports of Zika virus (1947 to February 2016) broken down by country (yellow shading) and an ensemble niche model built from occurrence data (red shading). Our model correspond well to shaded countries, with only minor discrepancies (Paraguay, the Central African Republic; a single case in Egypt in the 1950s), We emphasize that displaying cases at country resolution overstates the distribution of the virus, especially in the Americas (for example, Alaska, a point of significant concern given Messina *et al*.’s presentation of their niche model in terms of “highly suitable” countries with broad geographic expanse like the United States, China, and Argentina.

Our final Zika model combines seven methods with a variable set chosen from bioclimatic variables and a vegetation index to minimize predictor covariance. The ensemble model performs very well (AUC = 0.993; Fig 1), to a degree that resembles overfitting but is in fact driven by the strength of the ensemble modeling approach (which preferentially weights the best models *across* iterations, minimizing the error associated with any given high-performing iteration). The model strongly matches most occurrences including the hotspots of Brazilian microcephaly. It also predicts additional regions where Zika is so far unrecorded, but where further inquiry may be desired (in particular, Southern Sudan and the northern coast of Australia). Our model indicates that certain occurrences, like the 1954 report from Egypt and almost all North American cases, are likely outside the stable transmission niche (i.e., persistent over time) of the virus (*sensu* [**36**]). Moreover, we note that visual presentation of cases–or, of ecological niche models–at the country level may make the range of the virus appear far larger than our models suggest (see Fig 1).

**Fig 2:**
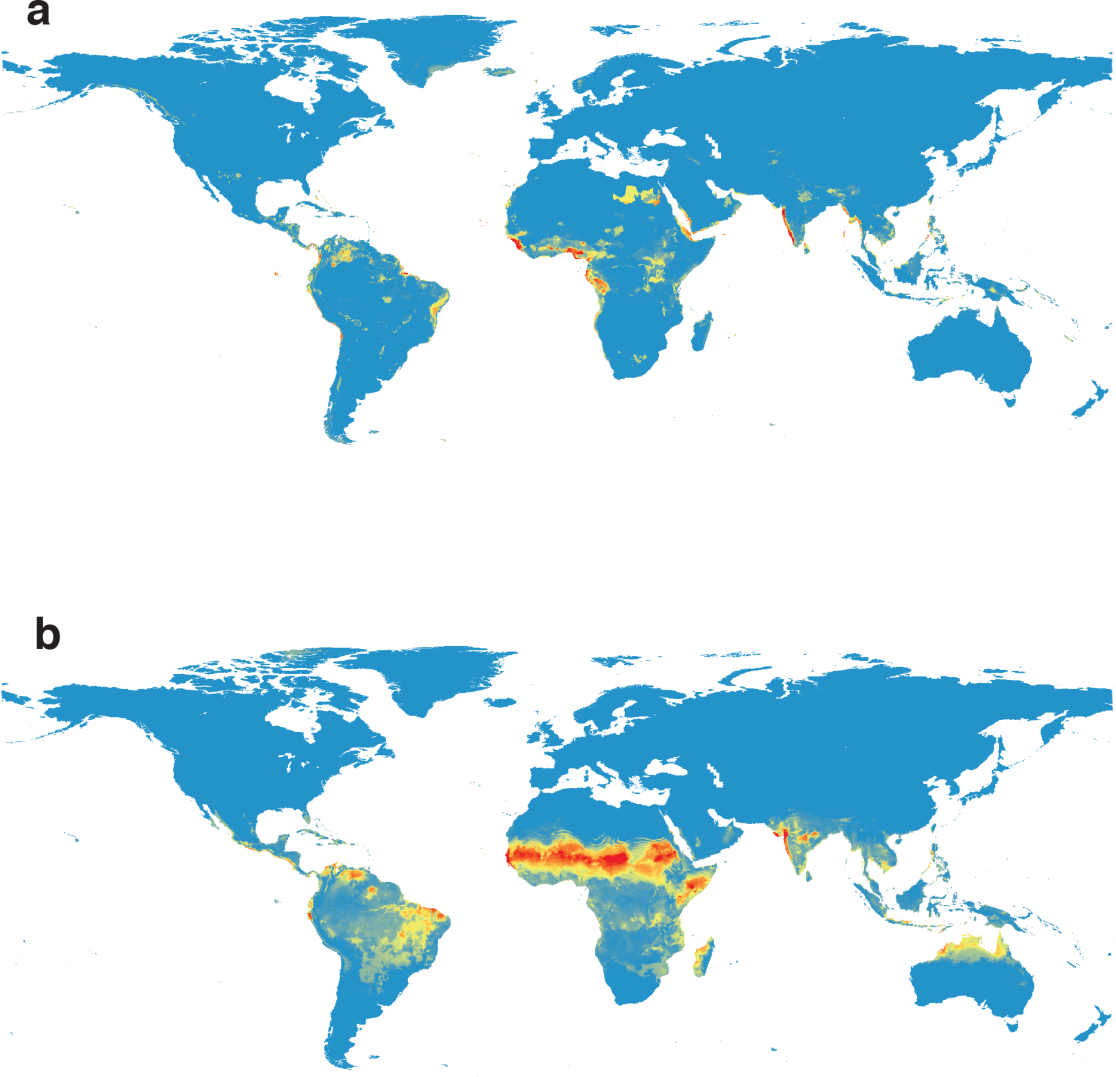
Geographical cross validation of (a) the sub-model built from occurrences on the African continent (n = 27) as projected upon the global climate space and (b) the sub-model built from occurrences on the Asian continent (n = 33) projected at the global scale.

Given the public health crisis posed by Zika, and the potential costs associated with underpredicting the extent of the current outbreak, we pay special attention to evaluating the sensitivity of our models to variations in our preliminary dataset. Historical geographical data on cases in the Americas are lacking, given the recent introduction of the virus, and the routes and drivers of transmission involved in that outbreak are uncertain, preventing meaningful cross-validation of models of the current outbreak with our Old World model. However, it is worth noting that recent phylogenetic work suggests a deep phylogenetic division between African and Asian strains, the latter of which as a monophyletic group include the entire radiation through French Polynesia into current outbreak areas; [**11, 37**] to address the potential evidence that African and Asian strains of the virus may be ecologically distinct, we present models trained on each continent and projected globally as a basic sensitivity analysis (Fig 2).

The two models cross-validate weakly compared to the performance of the global model; driven by both the 50% reduction in sample size and the higher degree of aggregation of Asian occurrences, the two projected distributions are dramatically different. Despite the over-prediction of the Asian model in Africa and the possible overfitting of the African model, we emphasize that neither extreme scenario predicts any substantially greater range in North America than our main ensemble model. Moreover, our Asian model underpredicts but does predict two major hotspots of occurrence in Brazil, the Ceara/Rio Grande do Norte region and Roraima, both of which spatially correspond to hotspots of Zika according to the recent Faria *et al*. publication in *Science*, [**11**] adding further support to the model. Finally, despite low transferability between continents, both sub-models are well matched by our aggregated model in their native range, further supporting the accuracy and predictive power of our global projection.

**Fig 3:**
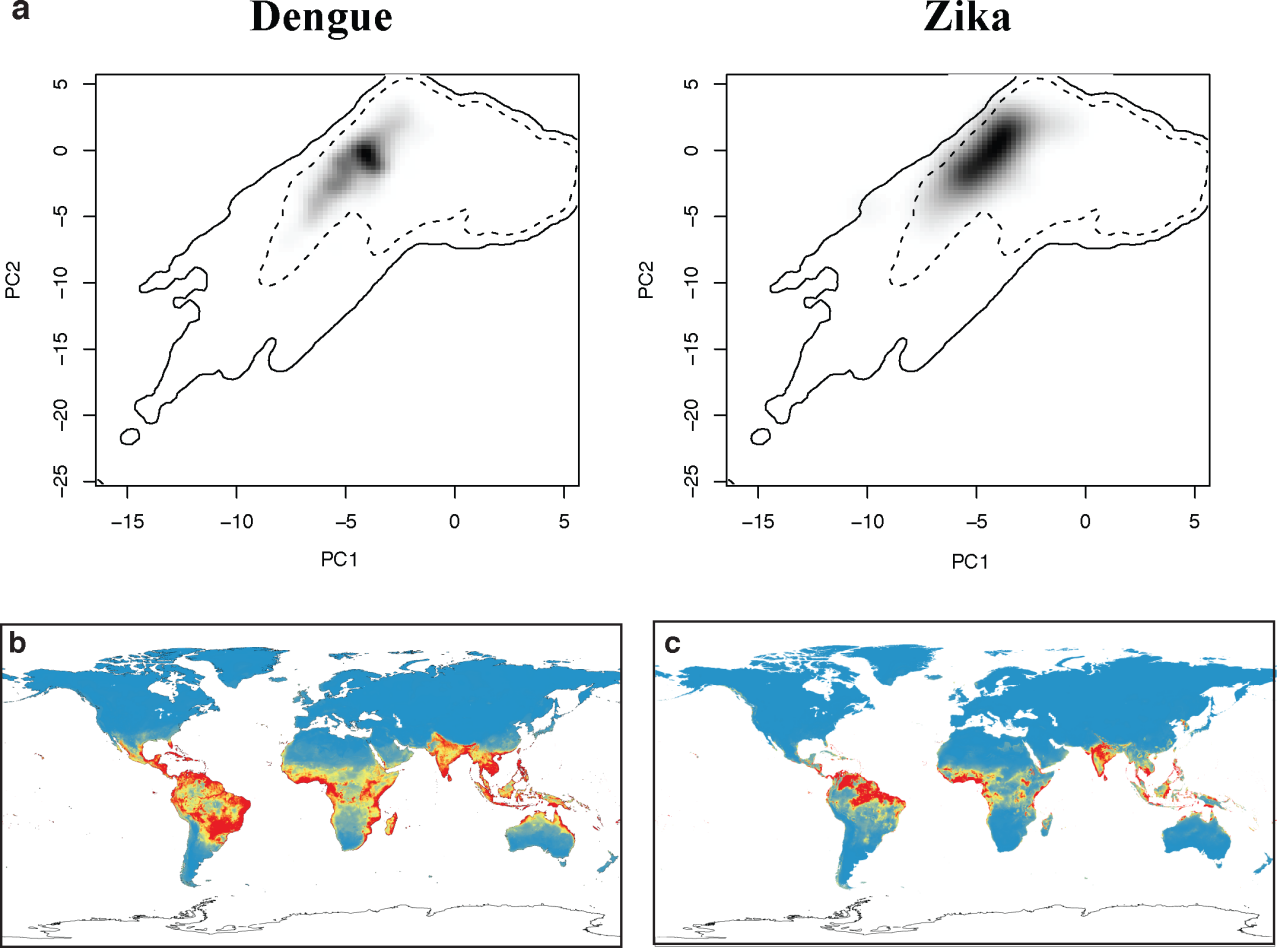
The ecological niche of Zika and dengue in principal component space (a). Solid and dashed lines are 100% and 50% boundaries for all environmental data, respectively. Despite apparent overlap in environmental niche space, the dissimilarity between the black shading in each principal component graph indicates statistically significant differences between the niches, evident in the projections of our niche models for dengue (b) and Zika (c).

Recently published work by Bogoch *et al*. [**38**] uses an ecological niche model for dengue as a proxy for the potential full distribution of ZIKV in the Americas, presenting findings in terms of potential seasonal vs. full-year transmission zones. While that approach has been effectively validated for dengue transmission in mosquitoes, using a model of one disease to represent the potential distribution of another emerging pathogen is only a placeholder, and is particularly concerning given the lack of evidence in our models that ZIKV and dengue have a similar niche breadth. [**39**] Comparing our niche models for dengue and ZIKV reveals that the two niches are significantly different (Schoener’s *D* = 0.176; *p* < 0.01; Fig 3). While the two occupy a similar region of global climate space, Zika is more strictly tropical than dengue, occupying regions with higher diurnal temperature fluctuations and seasonality of precipitation (Fig 3A).

**Fig 4:**
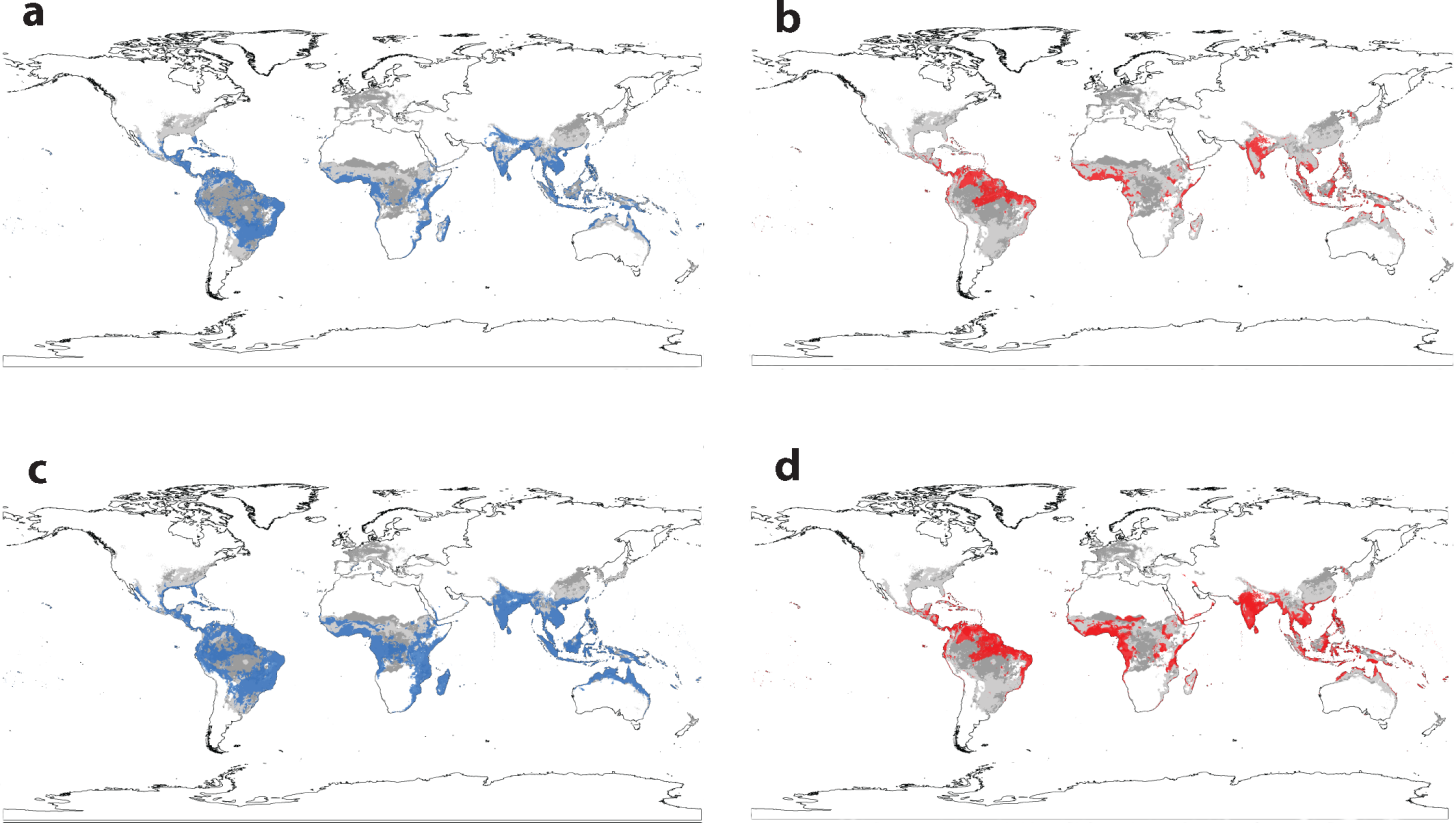
The estimated global distribution of Zika (red) and dengue (blue) based on current (a, b) and 2050 climate projections (c, d), compared against the current (light grey) and future distribution (dark grey) of all three mosquito vectors *Aedes aegypti*, *Ae*. *africanus* and *Ae*. *albopictus* (a-d).

Projecting niche models to the year 2050 suggests that expansion of Zika’s niche outside the tropics is an unlikely scenario, independent of vector availability (Fig 4). However, significant westward expansion in South America and eastward expansion in Africa implies that Zika may continue to emerge in the tropics. Moreover, our future projections for dengue (which strongly agree with previously published ones [**40**]) show an expansion out of the tropics that is not shared with Zika (Fig 4). These results call into question the applicability of dengue niche models used to project a significant future range for Zika in North America. [**38**]

**Fig 5:**
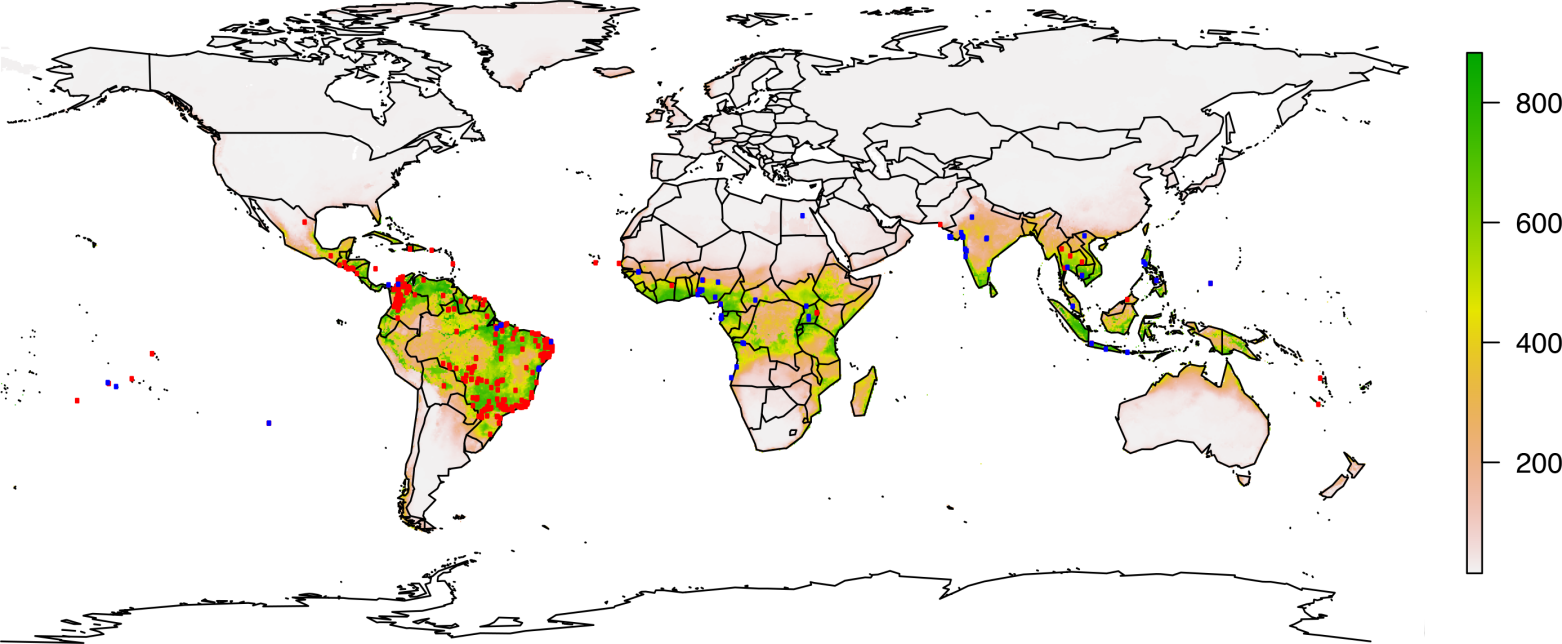
An updated ecological niche model incorporating aggregated global data, with Messina *et al*.’s full dataset (red) and ours (blue) against the updated weighted ensemble model.

Finally, we add a last layer of validation in the form of an analysis aggregating our and Messina *et al*.’s data, and include the results of an updated ensemble model in Fig 5 (as well as S6 and S7). Even with spatial thinning, that updated model is still heavily biased in favor of the South American occurrence data, which it predicts excellently, compared to a weaker fit in Africa and Asia. That accompanying loss of specificity is partly responsible for a lower AUC than our main model (AUC = 0.970) and the low TSS-based threshold (271, from 0 to 1000) that produces the substantially-greater predicted range shown in S7 Fig. The model does predict the current outbreak more effectively than ours, in particular better encompassing the southern half of Brazil where a surprising number of cases are clustered. But those southward expansions are accompanied by far less expansion above the equator in the Americas, and once again with the exception of the southernmost tip of Florida, there is no substantial predicted range in the United States, even along the Gulf Coast. If model discrepancies are attributed to evolutionary change and not to differences in model methods and specificity, those evolutionary changes seem to have done little to expand the North American niche of the virus (S8 Fig).

## Discussion

Ecological niche modeling has become one of the most generalized and useful parts of the streamlined response process for emerging infections. Recently published ecological niche models for Zika using MAXENT [**30**] and boosted regression trees [**29**] have resulted in somewhat conflicting results. Samy *et al*., using data exclusively from the range of the current outbreak, project autochthonous transmission in the southeastern United States, and potentially throughout the U.S. following regional outbreaks introduced by travelers. Their analysis incorporates socioeconomic factors into prediction, a valuable extra dimension we did not incorporate into our analysis; but the prediction of regions throughout the United States and most of the European continent as suitable based on only these criteria (i.e. despite lacking available vectors) seems uninformative except for the prediction of sexual outbreaks. Samy *et al*., however, conclude: “In Western Europe, ZIKV transmission risk is enhanced by travel times and connectivity to known transmission areas; as such, isolated autochthonous cases may occur at least seasonally when competent vector species are present.” [**30**] Messina *et al*. have a similar finding, based on a primarily ecological approach applied to 323 occurrences mostly from the New World; they map out most countries in the world as highly suitable, including the United States, with the conclusion that 2.17 billion people live in countries within Zika’s potential expanse. [**29**] These studies, being contemporaneous, do not refer to each other, and their conflicting results could render Zika forecasts unclear to the media and policymakers.

Interpreting conflicts between these models and those published here requires acknowledging three fundamental problems. First, differences in virulence between American and Asian strains of the virus may have changed the range limits. The niche of the vector-borne disease is manifest in its transmission and prevalence in mosquitoes (as well as humans and reservoirs), and increases in virulence could change the threshold of habitat suitability manifest in range limits. Without comparative work using updated data in Samy *et al*. and Messina *et al*.’s papers, equal support exists for our differences being attributable to methodological discrepancies or to a difference between Asian and American strains. But in the preliminary analysis we present in the supplementary information, incorporating data from the New World does not substantially expand projections in the United States (though a greater region of Brazil is predicted); and we believe a combination of evolutionary shifts and methodological differences is likely the most parsimonious explanation for differing results.

Second, we acknowledge the untested possibility that Zika has been expanding in its range since discovery in the 1940s (though, the virus was soon recorded in Borneo and Vietnam in the 1950s [**23**]), which would also decrease both the accuracy of our models in that region, and their power in the New World compared to the models published in the other two studies. Testing that possibility using our data broken down by time periods would be strongly statistically biased by the non-random element of viral discovery in different tropical countries, a factor for which it would be nearly impossible to control. Phylogenetic evidence has placed the introduction in the Americas within the last decade [**11**], but the age of divergence between Zika and closely related viruses like Japanese and St. Louis Encephalitis Viruses is less certain. Improving phylogenetic evidence based on updated Old World genomes in the coming years is a far more appropriate methodology for testing different biogeographic theories within that region.

Third and finally, we acknowledge the possibility that dispersal limitations have changed between the Old and New World, in such a way that the present expansion of Zika is not the emergence of novel niche space but the manifestation of hidden plasticity. This possibility is troubling from a public health perspective: if Zika’s niche is simply more expansive than current data/models capture, its geographic expansion could progress much further than we predict. This problem is fundamental to all predictive models applied to biological invasions, but Broenniman & Guisan [**35**] suggest that combining data from the native and invasive range maximizes the utility of ENMs in these scenarios. In our combined model we find evidence for subtle differences, especially in South America, but our findings remain sound with respect to the boundaries of transmission in North America. In any niche modeling study, there is always the possibility for error by omission; but we find no evidence that this has occurred in our study.

The dynamics of arboviruses at the range margins of their vectors are complex. In the case of dengue, the distribution of the virus in the United States (and elsewhere in temperate regions) remains more constrained than the range of its vectors. Our paper tests and rejects the hypothesis that predictions of Zika will occupy the entire niche of *Aedes* populations in North America, disagreeing with the two recently published niche model studies. Our models imply a similar constraint on Zika transmission to that of dengue if not a more pronounced one, and owing to the complexities surrounding transmission dynamics at the edges of suitable ranges, [**41**] the potential existence of Zika in even the southernmost parts of Florida [**42**] may not sustain autochthonous Zika transmission indefinitely. Making more specific predictions within Florida can be done through ecological niche models, but is likely more appropriately achieved through conventional epidemiological models that explicitly model vector abundance, biting rates and phenology.

Our models find an ecological nonequivalence of Zika and dengue, and suggest that the niche of the virus in both Africa and Asia is far narrower than what other models project based on current outbreak data or based on knowledge of dengue’s spread. We reject our first hypothesis, but based on the occurrence of Zika cases outside our predicted suitable range for the virus, we cannot eliminate our second hypothesis that the 2016 Zika outbreak may be in ephemeral, rather than stable, parts of the Zika transmission niche due to episodic climatic conditions. Specifically, El Niño Southern Oscillation (ENSO) events drive outbreaks of dengue in the Americas and in Southeast Asia, [**43**] and Paz *et al*. [**12**] have conjectured that the 2015 ENSO event could have contributed to the severity of the ZIKV outbreak in North and Central America (in response to Bogoch *et al*. [**38**]). While wind-dispersed mosquitoes carrying infections can be responsible for the introduction of diseases to new regions, [**44**] reported cases in the United States have all been contracted sexually or while traveling abroad to regions with endemic outbreaks, further supporting the tropical constraint hypothesis. However, in the second hypothesis scenario, the rapid expansion during the current outbreak beyond the boundaries of the stable transmission niche is unlikely to be followed by naturalization of the pathogen in the United States in the future, except perhaps in the southernmost tip of Florida. While ecological niche models relate occurrence to climate, drivers of disease may operate at the temporal scale of weather, and we suggest further analyses of a different methodology are necessary to confirm or reject the potential contribution of El Nino or anomalous storms to Zika’s expansion.

In the case of our third hypothesis, if alternative modeling efforts based on data from the Americas are evidence that the niche of the American strain of the virus has broadened, it is possible that mutations allowing increased virulence or changing transmission dynamics have occurred (and that weather events have not driven the severity of the current outbreak). From the results of our supplementary analysis using aggregated global data, we continue to treat the third hypothesis as a hypothesis for which there may be weak evidence. But we suggest it cannot be rejected or accepted confidently unless alternative hypotheses are eliminated and more evidence is collected–in particular, empirical data demonstrating or failing to find differences in transmission dynamics or virulence between the native Asian virus and its invasive descendant (rather than global comparisons and cross-validations of different ecological niche models).

Our models nevertheless suggest it could be premature to expect Zika naturalization as a widespread eventuality in North America, as other models have forecasted. Without more definitive information on the basic biology of Zika, however, the confidence with which niche models can forecast pandemics is limited. In particular, we also draw attention to recent evidence suggesting Zika persistence may depend on wildlife reservoirs in addition to human hosts and mosquitoes. Primates have been suggested as the primary candidate clade because the Zika flavivirus was first isolated in a rhesus macaque in the Zika Forest in Uganda. But as rhesus macaques do not occur on the African continent, and were captive there for inoculation experiments, the primate reservoir hypothesis remains unsupported. A 2015 case of an Australian presumed to have contracted Zika from a monkey bite while traveling in Indonesia, however, indicates that primates may transmit the virus directly. [**9**] Additionally, antibodies against Zika have been observed in several rodent and livestock species in Pakistan, [**45**] as well as several large mammal species, including orangutans, zebras, and elephants. [**46**] The potential for any North American wildlife species to play host to Zika is, at the present time, entirely unknown, and the emergence of novel amplification hosts (which may allow the virus to proliferate above the host density threshold in vectors in regions otherwise unsuitable for sustained transmission) could potentially expand the suitable range margins of Zika infection on a global scale.

From the results of our model we find strong evidence for the hypothesis that the global threat of a specifically vector-borne Zika pandemic, though devastating, may be most acute in the tropics; and we find that the evidence of future North American transmission in the literature is not unequivocal. However, we concur with the scientific majority that sexual transmission of Zika infections may still facilitate a significant outbreak in the United States and other previously unsuitable regions, particularly under evolutionary processes that select for the most directly transmissible strains of pathogens. [**47**] A case of sexual transmission in Texas has been suspected in the 2016 outbreak, and two previous reports of likely sexual transmission of ZIKV occurred in 2011 and 2015. [**5, 48**] Even if the Zika cases in the United States represent a rare spillover outside of the mosquito-borne viral niche, sexual transmission could create a new, unbounded niche in which the virus could spread. We draw attention to the potential parallels with simian and human immunodeficiency virus (SIV/HIV), for which a sexually transmitted pandemic has overshadowed the zoonotic origin of the disease. [**49**] With Zika’s asymptomatic presentation and the overall confusion surrounding its basic biology and transmission modes, we caution that its potential for severe sexually-transmitted outbreaks cannot be overlooked in the coming months.

To address the broader community of modelers and ecologists involved in the Zika intervention, we conclude with a final cautionary note. The consequences of under-predicting an outbreak’s potential distribution are obvious and our results are phrased cautiously as a result. But there are also economic and social consequences to over-predicting the potential distribution, especially in the United States. The response to Zika is necessarily political and consequently involves the division of resources between domestic preparedness and international relief; while new tools are being developed to help allocate funds efficiently based on epidemiological principles (we particularly highlight the work of Alfaro-Murillo *et al*. [**50**]), global overestimation of the virus’s trajectory could vastly reduce the power of those methods.

Models like those of Messina *et al*. and Samy *et al*. that predict substantial Zika expansion in the United States, and in the case of the former suggest Zika could threaten up to 2.17 billion people, contribute (independent of accuracy) to fear of an American pandemic. This prediction necessarily diverts funding away from relief efforts in Brazil and other affected countries in Latin America, increasing the probability of traveler infections feeding sexual outbreaks in the U.S.; and further reduces the credibility and impact of the American foreign response to Zika by mobilizing potentially-unnecessary domestic responses. At the time of writing, the Zika Vector Control Act passed by the U.S. House of Representatives weakens permit requirements for spraying pesticides near bodies of water without reallocating any funding for Zika interventions; and preventative efforts in New York City alone will cost $21 million to trap mosquitoes and hire epidemiological experts, with other cities outside our predicted range investing in preparation and vector control to similar degrees. Voices of scientific authority contributing to fear in the United States can substantially impact the political response to Zika, and it serves future modeling efforts to be as accurate, cautious, and objective as possible in the information and statistics that underpin media and policy conversations. But even more importantly, scientific teams with different approaches and data must work collaboratively to interpret the discrepancies between their results and to build an unbiased scientific consensus that is accessible to the public.

## Supporting Information

S1 Table Global occurrence database for Zika virus. A dataset containing the country, locality string used for geo‐referencing, latitude and longitude (in decimal degrees), uncertainty radius, comments, and the reference from which the data were obtained, followed by an exhaustive reference list.
(PDF)

S2 Table Zika full variable set preliminary model variable importance. Variable contributions are based on one preliminary run with 20 variables and 10 candidate models.
(PDF)

S3 Table Dengue full variable set preliminary model variable importance. Variable contributions are based on one preliminary run with 20 variables and 10 candidate models.
(PDF)

S4 Table *Aedes aegypti* full variable set preliminary model variable importance. Variable contributions are based on one preliminary run with 20 variables and 10 candidate models.
(PDF)

S5 Table *Aedes africanus* full variable set preliminary model variable importance. Variable contributions are based on one preliminary run with 20 variables and 10 candidate models.
(PDF)

S6 Table *Aedes albopictus* full variable set preliminary model variable importance. Variable contributions are based on one preliminary run with 20 variables and 10 candidate models.
(PDF)

S7 Table AUC of ten models for five species (with reduced variable sets). Bolded models were shown in the final models. Updated Zika model incorporating New World outbreak data included as “ZIKV+”.
(PDF)

S8 Table Zika final model variable importances. The final ensemble model includes seven modeling methods using sixteen variables, each run for 10 iterations.
(PDF)

S9 Table Dengue final model variable importances. The final ensemble model includes eight modeling methods using sixteen variables, each run for 10 iterations.
(PDF)

S10 Table *Aedes aegypti* final model variable importances. The final ensemble model includes eight modeling methods using sixteen variables, each run for 10 iterations.
(PDF)

S11 Table *Aedes africanus* final model variable importances. The final ensemble model includes eight modeling methods using sixteen variables, each run for 10 iterations.
(PDF)

S12 Table *Aedes albopictus* final model variable importances. The final ensemble model includes eight modeling methods using sixteen variables, each run for 10 iterations.
(PDF)

S13 Table Variable importance in supplementary ZIKV+ model. (PDF)

S1 Fig Final ensemble model for Zika virus. (TIF)

S2 Fig Final ensemble model for dengue fever. (TIF)

S3 Fig Final ensemble model for *Aedes aegypti*. (TIF)

S4 Fig Final ensemble model for *Aedes africanus*. (TIF)

S5 Fig Final ensemble model for *Aedes albopictus*. (TIF)

S6 Fig Expanded niche model with global data coverage. (TIF)

S7 Fig Expanded niche model with threshold. (TIF)

S8 Fig Niche Overlap Analysis between Dengue and Global Zika Database. In the equivalency test, we find significant evidence for differences (Schoener’s D = 0.295; p = 0.004).
(TIF)
