# Supplementary material for: An Ecological Assessment of the Pandemic Threat of Zika Virus
Source: PLoS Negl Trop Dis. 2016 Aug 26;10(8):e0004968. doi: 10.1371/journal.pntd.0004968 (PMC5001720; doi:10.1371/journal.pntd.0004968)
Supplement: S1 Table — A dataset containing the country, locality string used for geo‐referencing, latitude and longitude (in decimal degrees), uncertainty radius, comments, and the reference from which the data were obtained, followed by an exhaustive reference list. (PDF) [file pntd.0004968.s001.pdf]

Table S1. A spatially explicit occurrence database for Zika virus

Country	Locality String	GeoLat	GeoLong	Uncert. (m)	Comments	Study
**Angola**	Maquela do Zombo	-6.050078	15.108851	4083		[1]
**Angola**	Beù	-6.231823	15.471298	21525		[1]
**Angola**	Lobito	-12.371888	13.562743	5058		[1]
**Angola**	Mocamedes	-15.203731	12.153252	3465		[1]
**Brazil**	Santa Helena Hospital, Camacari, Bahia	-12.700504	-38.332377	NA	Exact location of hospital	[2]
**Brazil**	Salvador	-12.983333	-38.516667	18306	Uncertain (numerous candidates)	[3]
**Brazil**	Belém	-1.453613	-48.478676	6942		[4]
**Brazil**	Benevides, Pará	-1.359566	-48.249232	3199		[5]
**Brazil**	Cametá	-2.25	-49.5	4803		[4]
**Brazil**	Natal, Rio Grande do Norte	-5.795001	-35.208891		05°47’42”S, 35°12’32”O	[6]
**Cambodia**	Kampong Speu Province	11.561896	104.34157	60463		[7]
**Cameroon**	Provincial Hospital Annex and Mount Mary Health Centre in Buea & Cameroon Development Corporation Central Clinic, Tiko, Fako Division, Cameroon	4.075185	9.3699	3573		[8]
**Central African Republic**	Bozo	5.140642	18.478947	579		[9]
**Chile**	Easter Island	-27.116667	-109.366667	13845		[10]
**Colombia**	Sincelejo	9.304722	-75.397778	3645		[11]
**Egypt**	Elwan	27.269	30.88			[12]
**French Polynesia**	Bora Bora	-16.4944	-151.7364	NA		[13]
**French Polynesia**	French Polynesia				Too broad a region	[10]
**French Polynesia**	Centre Hospitalier du Taaone	-17.528796	-149.546573	NA		[14]
**French Polynesia**	Tahiti				(Duplicate with another)	[15]

**Gabon**	Cocobeach	1.000186	9.582285	1614		[16]
**Gabon**	Diba-Diba, Libreville	0.383333	9.45	10421		[16]
**Gabon**	Nzeng-Ayong, Libreville	0.421697	9.478728	1304		[16]
**Gabon**	PK6, Libreville	0.405025	9.479887	724		[16]
**Gabon**	PK8, Libreville	0.410089	9.49259	687		[16]
**Gabon**	Alenkiri, Libreville	0.304433	9.509842	2041		[16]
**India**	Paud, Poona, Bombay	18.52404	73.615322	5793		[17]
**India**	Khandala, Poona, Bombay	18.059182	74.015993	5692		[17]
**India**	Rasalpur, East Khandesh, Bombay	26.900684	75.424156	2809	Corrected to: Asalpur	[17]
**India**	Zagadia, Broach, Bombay	21.714349	73.15101	3454	Corrected to: Jhagadia	[17]
**India**	Ankleswar, Broach, Bombay	21.632356	72.990015	6147	Corrected to: Ankleshwar	[17]
**India**	Sajad, Broach, Bombay					[17]
**India**	Jambusar, Broach, Bombay	22.052359	72.800741	2145		[17]
**India**	Broach, Broach, Bombay	21.694821	72.980501	7010	Corrected to: Bharuch	[17]
**India**	Bareja, Ahmedabad, Bombay	22.848628	72.591375	1434		[17]
**India**	Modhwada, Porbander, Saurashtra	21.816703	69.522944	NA	Corrected to: Modhvada	[17]
**India**	Porbander, Porbander, Saurashtra	21.641346	69.600868	5418	Corrected to: Porbandar	[17]
**India**	Kutiyana, Sorath, Saurashtra	21.624099	69.984937	1690	Sorath outdated	[17]
**India**	Ranavav, Sorath, Saurashtra	21.687339	69.74485	3073	Sorath outdated	[17]
**India**	Ramtek, Nagpur, Madhya Pradesh	21.395514	79.327017	3556	Sorath outdated	[17]
**India**	Unrer, Nagpur, Madhya Pradesh				Could not be located	[17]
**India**	Kamptee, Nagpur, Madhya Pradesh	21.217137	79.194531	3531		[17]
**India**	Mehbubnagar, Mehbubnagar, Hyderabad	16.567033	73.737634	3269	Corrected to Mahabubnagar	[17]

**India**	Tiruvellore, Chingleput, Madras	13.143745	79.905682	3613	Corrected to: Tiruvallur	[17]
**Indonesia**	Jakarta	-6.2182	106.8584	23109		[18]
**Indonesia**	Tegalyoso Hospital, Klaten, Central Java	-7.705833	110.606389	4681		[19]
**Indonesia**	Lombok	-8.554324	116.293132	58689		[20]
**Malaysia**	Bentong, West Central Mal.	3.5316	101.9038	7068		[21]
**Micronesia**	Yap Island	9.533149	138.11617	NA		[22]
**Micronesia**	Yap Island				(Duplicate with another)	[23]
**Micronesia**	Federated States of Micronesia				Too broad a region	[10]
**New Caledonia**	New Caledonia				Too broad a region	[10]
**Nigeria**	Kainji Lake Basin	10.394167	4.555556	64023		[24]
**Nigeria**	Ikeja near Lagos	6.594222	3.337475	4019		[25]
**Nigeria**	Oshogbo, Oyo State	7.781975	4.549158	6627		[26]
**Nigeria**	Igbo-Ora, Oyo State	7.742222	3.804444	1785		[26]
**Nigeria**	Ibadan, Oyo State	7.387778	3.896389	17815		[26]
**Nigeria**	Oyo, Oyo State	7.840178	3.931335	12843		[26]
**Nigeria**	Tagwe (Miango), Jos Plateau	9.85	8.683333	7840		[27]
**Nigeria**	Afikpo Division	5.893121	7.937353	5781	Approximate	[28]
**Panama**	Utupo	9.137909	-77.930758	1266		[29]
**Phillipines**	Cebu City	10.3	123.9	16394		[30]
**Phillipines**	Sapangbato Field	15.175368	120.513715	3036		[31]
**Phillipines**	Negritos Jungle					[31]
**Phillipines**	Manila Urban	14.5833	121	25288		[31]
**Senegal**	Ke´dougou region, SE Sen.	12.550012	-12.183355	NA	12°33' N, 12°11' W	[32]
**Senegal**	Bandafassi, SE Sen.	12.5406	-12.3077	3951		[33]
**Thailand**	Bangkok	13.753979	100.501444	47411		[34]
**Uganda**	Zika Forest	0.124167	32.525556	NA		[35]
**Uganda**	Madi District, Laropi, Dufile	3.560679	31.880368	10138		[36]
**Uganda**	Mengo B, Luwero, Bukomero,	0.842124	32.492496	2445		[36]

	Ntenjeru and Nakasongol					
**Vanuata**	Vanuatu				Too broad a region	[37]
**Vietnam**	Tonkin/Tong-King, Red River Delta	22	105	2776		[34]

Literature Cited

[1] Kokernot, R. H., Casaca, V. M. R., Weinbren, M. P. & McIntosh, B. M. Survey for antibodies against arthropod-borne viruses in the sera of indigenous residents of Angola. *T. Roy. Soc. Trop. Med. H.* **59**, 563-570 (1965).

[2] Campos, G.S., Bandeira, A.C. & Sardi, S.I. Zika virus outbreak, Bahia, Brazil. *Emerg. Infect. Dis.* **21**, 1885 (2015).

[3] Cardoso, C.W., *et al.* Outbreak of Exanthematous Illness Associated with Zika, Chikungunya, and Dengue Viruses, Salvador, Brazil. *Emerg. Infect. Dis.* **21**, 2274–2276 (2015).

[4] Causey, O.R., & Theiler, M. Virus antibody survey on sera of residents of the Amazon Valley in Brazil. *Am. J. Trop. Med. Hyg.* **7**, 36-41 (1958).

[5] Pan American Health Organization. Epidemiological Alert: Neurological syndrome, congenital malformations, and Zika virus infection. Implications for public health in the Americas. 1 December 2015. http://www.paho.org/hq/index.php?option=com_docman&task=doc_download&Itemid=&gid=32405&lang=en. Accessed 2/10/2016

[6] Zanluca, C., *et al.* First report of autochthonous transmission of Zika virus in Brazil. *Mem. I. Oswaldo Cruz* **110**, 569-572 (2015).

[7] Heang, V., *et al*. Zika virus infection, Cambodia, 2010. *Emerg. Infect. Dis*. **18**, 349 (2012).

[8] Fokam, E. B., *et al*. Silent circulation of arboviruses in Cameroon. *E. Afr. Med. J.* **87**, 262-268 (2010).

[9] Berthet, N., *et al.* Molecular Characterization of Three Zika Flaviviruses, Obtained from Sylvatic Mosquitoes in the Central African Republic. *Vector Borne Zoonotic Dis* **14,** 862-865 (2014).

[10] Musso, D., Nilles, E.J. & Cao-Lormeau, V.M. Rapid spread of emerging Zika virus in the Pacific area. *Clin. Microbiol. Infec*. **20,** O595-O596 (2014).

[11] Camacho E, Paternina-Gomez M, Blanco PJ, Osorio JE, Aliota MT. Detection of autochthonous Zika virus transmission in Sincelejo, Colombia. *Emerg Infect Dis.* 2016 May. http://dx.doi.org/10.3201/eid2205.160023

[12] Smithburn, K. C., *et al.* Immunity to certain arthropod-borne viruses among indigenous residents of Egypt. *Am. J. Trop. Med. H*. **3**, 9-18 (1954).

[13] Kutsuna, S., *et al*. Two cases of Zika fever imported from French Polynesia to Japan, December 2013 to January 2014. *Euro. Surveill.* 19, 1-5 (2014).

[14] Cao-Lormeau, V.M. *et al*. Zika Virus, French Polynesia, South Pacific, 2013. DOI: http://dx.doi.org/10.3201/eid2006.140138

[15] Wæhre T., Maagard, A., Tappe D., Cadar D. & Schmidt-Chanasit, J. Zika Virus Infection after Travel to Tahiti, December 2013. *Emerg. Infect. Dis*. **20**, 1412-1414 (2014).

[16] Grard, G., *et al.* Zika virus in Gabon (Central Africa)–2007: a new threat from Aedes albopictus. *PLoS Negl. Trop. Dis*. **8**, e2681 (2014).

[17] Smithburn, K. C., Kerr, J.A. & Gatne, P.B. Neutralizing antibodies against certain viruses in the sera of residents of India. *J. Immunol.* **72**, 248-257 (1954).

[18] Kwong, J.C., Druce, J.D. & Leder, K. Zika virus infection acquired during brief travel to Indonesia. *Am. J. Trop. Med. H*. **89**, 516-517 (2013).

[19] Olson, J. G. & Ksiazek, T.G. Zika virus, a cause of fever in Central Java, Indonesia. *T. Roy. Soc. Trop. Med. H.* **75**, 389-393 (1981).

[20] Olson, J. G., *et al.* A survey for arboviral antibodies in sera of humans and animals in Lombok, Republic of Indonesia. *Ann. Trop. Med. Parasit.* **77**, 131-137 (1983).

[21] Marchette, N. J., Garcia, R. & Rudnick, A. Isolation of Zika virus from Aedes aegypti mosquitoes in Malaysia. *Am. J. Trop. Med. H*. **18**, 411-415 (1969).

[22] Duffy, M.R., *et al*. Zika virus outbreak on Yap Island, federated states of Micronesia. *N. Eng. J. Med.* **360**, 2536-2543 (2009).

[23] Lanciotti, R.S., *et al*. Genetic and serologic properties of Zika virus associated with an epidemic, Yap State, Micronesia, 2007. *Emerg. Infect. Dis*. **14**, 1232 (2008).

[24] Adekolu-John, E. O. & Fagbami, A.H. Arthropod-borne virus antibodies in sera of residents of Kainji Lake Basin, Nigeria 1980. *T. Roy. Soc. Trop. Med. H*. **77**, 149-151(1983).

[25] Boorman, J. P. T., & Porterfield, J.S. A simple technique for infection of mosquitoes with viruses transmission of Zika virus. *T. Roy. Soc. Trop. Med. H*. **50**, 238-242 (1956).

[26] Fagbami, A. H. Zika virus infections in Nigeria: virological and seroepidemiological investigations in Oyo State. *J. Hygiene - Cambridge* **83**, 213-219 (1979).

[27] Lee, V. H., & Moore, D.L. Vectors of the 1969 yellow fever epidemic on the Jos Plateau, Nigeria. *Bull. World Health Organ*. **46**, 669 (1972).

[28] Macnamara, F.N. Zika virus: a report on three cases of human infection during an epidemic of jaundice in Nigeria. *T. Roy. Soc. Trop. Med. H*. **48**, 139-145 (1954).

[29] http://www.minsa.gob.pa/noticia/ministerio-de-salud-comunica-la-poblacion-panamena-sobre-el-virus-zika

[30] Alera, M.T., *et al*. Zika virus infection, Philippines, 2012. *Emerg. Infect. Dis*. **21**, 722 (2015).

[31] Hammon, W., Schrack, W.D. & Sather, G.E. Serological Survey for Arthropod-Borne Virus Infections in the Philippines. *Am. J. Trop. Med. Hyg.* **7**, 323-328 (1958).

[32] Diallo, D., *et al*. Zika virus emergence in mosquitoes in southeastern Senegal, 2011. PloS one **9**, e109442 (2014).

[33] Foy, B.D., *et al*. Probable non–vector-borne transmission of Zika virus, Colorado, USA. *Emerg. Infect. Dis*. **17**, 880 (2011).

[34] Pond, W.L. Arthropod-borne virus antibodies in sera from residents of South-East Asia. *T. Roy. Soc. Trop. Med. H*. **57**, 364-371 (1963).

[35] Dick, G. W. A., Kitchen, S.F. & Haddow, A.J.. Zika virus (I). Isolations and serological specificity. *T. Roy. Soc. Trop. Med. H*. **46**, 509-520 (1952).

[36] Henderson, B. E., Kirya, G.B. & Hewitt, L.E. Serological survey for arboviruses in Uganda, 1967-69. *Bull. World Health Organ*. **42**, 797 (1970).

[37] Musso, D., Cao-Lormeau, V.M. & Gubler, D.J.. Zika virus: following the path of dengue and chikungunya? *Lancet* **386**, 243-244 (2015).
