# Supplementary material for: An Ecological Assessment of the Pandemic Threat of Zika Virus
Source: PLoS Negl Trop Dis. 2016 Aug 26;10(8):e0004968. doi: 10.1371/journal.pntd.0004968 (PMC5001720; doi:10.1371/journal.pntd.0004968)
Supplement: S2 Table — Variable contributions are based on one preliminary run with 20 variables and 10 candidate models. (PDF) [file pntd.0004968.s002.pdf]

Table S2. Zika full variable set preliminary model variable importance

	GLM	GBM	GAM	CTA	ANN	SRE	FDA	MARS	RF	MAXENT
**bio1**	0.573	0.009	0.727	0	0.089	0.385	0.001	0	0.022	0.294
**bio2**	0.896	0.006	0.491	0	0.072	0.361	0	0	0.013	0.546
**bio3**	0.841	0.015	0.46	0	0.14	0.286	0.056	0	0.025	0.215
**bio4**	1	0	0.552	0	0.527	0.372	0	0	0.014	0.06
**bio5**	0	0.003	0.659	0	0.421	0.231	0	0	0.014	0.487
**bio6**	0.611	0.205	0	0.935	0.218	0.463	0.468	0.708	0.064	0.177
**bio7**	0	0.031	0.444	0	0.62	0.296	0.151	0.343	0.04	0.263
**bio8**	0.219	0	0.439	0	0.211	0.412	0	0	0.007	0
**bio9**	0	0.009	0.625	0	0.012	0.302	0	0	0.014	0
**bio10**	1	0.002	0.698	0	0.036	0.336	0	0.266	0.012	0
**bio11**	0	0.019	1	0	0.155	0.378	0	0	0.059	0.076
**bio12**	0	0.019	0.191	0	0.453	0.106	0.188	0.348	0.029	0.126
**bio13**	0	0.092	0.204	0	0.759	0.211	1	0.738	0.066	0.534
**bio14**	0.589	0.003	0.377	0.211	0.303	0.013	0.514	0.065	0.003	0.302
**bio15**	0	0.047	0.201	0.558	0.001	0.068	0.073	0.125	0.017	0.533
**bio16**	0	0.009	0.18	0	0.537	0.155	0.618	0.786	0.028	0.031
**bio17**	0.588	0.003	0.501	0	0.345	0.018	0.572	0	0.008	0.464
**bio18**	0	0.002	0.044	0	0.331	0.145	0.054	0.184	0.006	0.178
**bio19**	0	0.008	0	0	0.173	0.059	0.114	0.286	0.007	0.307
**NDVI**	0	0.073	0.272	0	0.025	0.27	0.043	0.204	0.048	0.624
