# Supplementary material for: An Ecological Assessment of the Pandemic Threat of Zika Virus
Source: PLoS Negl Trop Dis. 2016 Aug 26;10(8):e0004968. doi: 10.1371/journal.pntd.0004968 (PMC5001720; doi:10.1371/journal.pntd.0004968)
Supplement: S3 Table — Variable contributions are based on one preliminary run with 20 variables and 10 candidate models. (PDF) [file pntd.0004968.s003.pdf]

Table S3. Dengue full variable set preliminary model variable importance

	GLM	GBM	GAM	CTA	ANN	SRE	FDA	MARS	RF	MAXENT
**bio1**	0.295	0.001	0.366	0.052	0.04	0.242	0	0	0.015	0.007
**bio2**	0	0.035	0.136	0.202	0.016	0.1	0.078	0.004	0.044	0.048
**bio3**	0.062	0	0.054	0.065	0.002	0.216	0.018	0	0.016	0.05
**bio4**	0.21	0.013	0.59	0.024	0.262	0.241	0	0	0.03	0.013
**bio5**	0.146	0	0.293	0.011	0.024	0.196	0	0.029	0.008	0.008
**bio6**	0.172	0.013	0.286	0.41	0.08	0.271	0.348	0.5	0.035	0.011
**bio7**	0.427	0.031	0.724	0.161	0.134	0.232	0.057	0.298	0.045	0.038
**bio8**	0.203	0.01	0.152	0.041	0.017	0.262	0.004	0.315	0.015	0.023
**bio9**	0.242	0	0.149	0.005	0.084	0.257	0	0	0.007	0.024
**bio10**	0.477	0	0.521	0.002	0.006	0.209	0.036	0.078	0.007	0.003
**bio11**	0.94	0.068	0.639	0.005	0.002	0.274	0.797	0.352	0.043	0.212
**bio12**	0.033	0	0.095	0.024	0.059	0.124	0.141	0	0.016	0.009
**bio13**	0.324	0.007	0.083	0.017	0.052	0.207	0.714	0.472	0.013	0.003
**bio14**	0.111	0.004	0.31	0.047	0.008	0.017	0.048	0.348	0.025	0.078
**bio15**	0.003	0.001	0.075	0.016	0.018	0.079	0.017	0	0.007	0.022
**bio16**	0.118	0	0.113	0.015	0.208	0.16	0	0.105	0.01	0.025
**bio17**	0.033	0.004	0.102	0.102	0.13	0.074	0	0.172	0.022	0.006
**bio18**	0.013	0.002	0.007	0.005	0.031	0.117	0.004	0	0.017	0.015
**bio19**	0.012	0.018	0.035	0.048	0.052	0.079	0.056	0.029	0.024	0.073
**NDVI**	0.073	0.032	0.075	0.105	0.016	0.198	0.067	0.039	0.035	0.099
