# Supplementary material for: An Ecological Assessment of the Pandemic Threat of Zika Virus
Source: PLoS Negl Trop Dis. 2016 Aug 26;10(8):e0004968. doi: 10.1371/journal.pntd.0004968 (PMC5001720; doi:10.1371/journal.pntd.0004968)
Supplement: S4 Table — Variable contributions are based on one preliminary run with 20 variables and 10 candidate models. (PDF) [file pntd.0004968.s004.pdf]

Table S5. *Aedes africanus* full variable set preliminary model variable importance

	GLM	GBM	GAM	CTA	ANN	SRE	FDA	MARS	RF	MAXENT
**bio1**	0.437	0.003	0.56	0.245	0.141	0.305	0.231	0.085	0.005	0
**bio2**	0	0.006	0.54	0.217	0.051	0.055	0	0	0.005	0.251
**bio3**	0	0.009	0.628	0	0.049	0.465	0	0	0.029	0.489
**bio4**	0.032	0.372	0.751	0.845	0.726	0.486	0	0.56	0.04	0.42
**bio5**	0	0	0.564	0	0.089	0.235	0	0	0.002	0.065
**bio6**	0	0.003	0.477	0	0.137	0.317	0.09	0.368	0.015	0.132
**bio7**	0	0.012	0.73	0.108	0.165	0.367	0	0.406	0.012	0.253
**bio8**	0	0.008	0.603	0	0.186	0.336	0.126	0.402	0.01	0
**bio9**	0.625	0.003	0.617	0	0.108	0.311	0	0.388	0.006	0
**bio10**	0.447	0.001	0.709	0	0.133	0.252	0	0.419	0.003	0.183
**bio11**	0	0.025	0.605	0.094	0.365	0.457	0.953	0.629	0.019	0.248
**bio12**	0.11	0.007	0.484	0.03	0.185	0.386	0.02	0.266	0.007	0.043
**bio13**	0.231	0.002	0.381	0	0.344	0.399	1	0.43	0.007	0.118
**bio14**	0.177	0.001	0.3	0	0.112	0.035	0.033	0	0.004	0.35
**bio15**	0.243	0.001	0.349	0	0.035	0.148	0	0	0.003	0.128
**bio16**	0	0.004	0.478	0	0.513	0.323	0.532	0.431	0.008	0.267
**bio17**	0	0.002	0.074	0	0.103	0.04	0	0	0.005	0.117
**bio18**	0.28	0.011	0.307	0.186	0.186	0.23	0.085	0.099	0.007	0.397
**bio19**	0.293	0.124	0.446	0.485	0.861	0.035	0.673	0.248	0.052	0.44
**NDVI**	0.286	0.04	0.468	0.218	0.066	0.175	0.088	0.073	0.02	0.201

Table S6. *Aedes albopictus* full variable set preliminary model variable importance

	GLM	GBM	GAM	CTA	ANN	SRE	FDA	MARS	RF	MAXENT
**bio1**	0.289	0	0.216	0.011	0.09	0.248	0.173	0.006	0.01	0.017
**bio2**	0.06	0.017	0.048	0.031	0.031	0.249	0.025	0.018	0.082	0.012
**bio3**	0.279	0.003	0.277	0.13	0	0.193	0.185	0.261	0.016	0.018
**bio4**	0.528	0	0.352	0.016	0.197	0.242	0	0	0.015	0.008
**bio5**	0.131	0	0.875	0.015	0.007	0.305	0.363	0.202	0.012	0.014
**bio6**	0.262	0	1	0	0.334	0.237	0.626	0.136	0.017	0.019
**bio7**	0.742	0.004	0.607	0.104	0.511	0.256	0.071	0.417	0.05	0.009
**bio8**	0.001	0.001	0.047	0	0.03	0.275	0	0	0.008	0.038
**bio9**	0.007	0	0.1	0	0.049	0.249	0	0	0.008	0.038
**bio10**	0.835	0.01	0.581	0	0.004	0.281	0.619	0.338	0.007	0.017
**bio11**	0.164	0	0.292	0	0.012	0.236	0.624	0.129	0.011	0.023
**bio12**	0.041	0.001	0.009	0.057	0.482	0.299	0	0	0.011	0.015
**bio13**	0.207	0.001	0.274	0	0.295	0.276	0	0	0.01	0
**bio14**	0.029	0.001	0.04	0.003	0.016	0.229	0.04	0.069	0.012	0.01
**bio15**	0.007	0	0.02	0.002	0.004	0.138	0	0	0.015	0.203
**bio16**	0	0	0.051	0.006	0.013	0.278	0	0	0.015	0.002
**bio17**	0.019	0.002	0.027	0	0.037	0.243	0.046	0.039	0.006	0.022
**bio18**	0.147	0.474	0.075	0.377	0.315	0.287	0.44	0.122	0.114	0.066
**bio19**	0.003	0.005	0.011	0	0.012	0.213	0.013	0.008	0.013	0.019
**NDVI**	0.027	0.009	0.021	0.024	0.008	0.26	0.026	0.032	0.008	0.006

Table S7. AUC of ten models for five species (with reduced variable sets). Bolded models were shown in the final models. Updated Zika model incorporating New World outbreak data included as "ZIKV+".

	GLM	GBM	GAM	CTA	ANN	SRE	FDA	MARS	RF	MAXENT
A.Aeg	**0.975**	**0.980**	**0.981**	**0.977**	**0.957**	0.855	**0.974**	**0.976**	**1.000**	0.930
A.Afr	**0.983**	**0.999**	**1.000**	**0.985**	**0.967**	0.837	**0.959**	**0.979**	**1.000**	0.739
A.Alb*	**0.919**	**0.942**	**0.938**	**0.945**	0.882	0.760	**0.923**	**0.930**	**1.000**	**0.940**
ZIKV	**0.934**	**0.975**	**0.968**	**0.920**	0.773	0.741	**0.934**	**0.938**	**1.000**	0.807
ZIKV+	**0.920**	**0.975**	**0.946**	**0.921**	--	--	**0.927**	**0.936**	**1.000**	--
DENG*	**0.919**	**0.942**	**0.938**	**0.945**	0.882	0.760	**0.923**	**0.930**	**1.000**	**0.940**

Table S8. Zika final model variable importances

	GLM	GBM	GAM	CTA	FDA	MARS	RF
**bio1**	0.608	0.014	0.654	0	0	0	0.026
**bio2**	0.919	0.006	0.738	0.068	0	0	0.019
**bio3**	0.774	0.02	0.481	0.178	0.1	0	0.029
**bio4**	1	0	0.329	0	0	0	0.017
**bio5**	0	0.006	0.708	0	0	0.279	0.015
**bio6**	0.626	0.211	0	0.879	0.46	0.803	0.125
**bio7**	0	0.033	0.563	0	0.268	0.354	0.051
**bio10**	1	0.003	0.792	0	0	0	0.014
**bio11**	0	0.018	1	0	0	0.033	0.047
**bio12**	0	0.022	0.104	0	0.227	0	0.028
**bio13**	0	0.109	0.075	0	0.462	0.873	0.116
**bio15**	0	0.046	0.207	0.323	0.071	0.295	0.026
**bio16**	0	0.01	0	0	0.153	0.506	0.043
**bio17**	0.258	0.008	0.183	0.094	0.208	0.211	0.013
**bio19**	0	0.01	0.02	0.201	0.062	0.326	0.008
**NDVI**	0	0.082	0.266	0	0.095	0.027	0.06

Table S9. Dengue final model variable importances

	GLM	GBM	GAM	CTA	FDA	MARS	RF	MAXENT
**bio1**	0.388	0.001	0.363	0.004	0.248	0	0.017	0
**bio2**	0.004	0.029	0.054	0.097	0	0	0.053	0.021
**bio4**	0.159	0.012	0.241	0.076	0	0.233	0.06	0.031
**bio6**	0.236	0.008	0.213	0	0.266	0	0.045	0.017
**bio7**	0.702	0.036	0.571	0.328	0.639	0.406	0.073	0.043
**bio8**	0.225	0.009	0.083	0.055	0.006	0.235	0.021	0.084
**bio9**	0.272	0	0.063	0.008	0	0.186	0.012	0.029
**bio10**	0.427	0	0.502	0.033	0.508	0.051	0.015	0.071
**bio11**	1	0.109	0.818	0.527	0.85	0.158	0.062	0.487
**bio13**	0.012	0.008	0.138	0.098	0.072	0.104	0.02	0.001
**bio14**	0.032	0.009	0.082	0.147	0.022	0.028	0.034	0.03
**bio15**	0	0.001	0.031	0.028	0	0	0.011	0.03
**bio16**	0	0	0.075	0.005	0	0	0.016	0.007
**bio18**	0.005	0.002	0.004	0.011	0	0	0.025	0.001
**bio19**	0.032	0.026	0.055	0.064	0.069	0.052	0.048	0.074
**NDVI**	0.095	0.042	0.085	0.17	0.081	0.113	0.05	0.052

Table S10. *Aedes aegypti* final model variable importances

	GLM	GBM	GAM	CTA	ANN	FDA	MARS	RF
**bio1**	0.276	0.01	0.462	0.012	0.019	0.212	0.024	0.02
**bio2**	0.13	0.011	0.05	0.234	0.023	0	0	0.083
**bio3**	0.372	0.005	0.148	0.048	0.008	0.102	0.078	0.018
**bio4**	0.82	0.001	0.198	0.026	0.048	0	0	0.02
**bio5**	0.273	0	0.178	0.012	0.064	0.215	0	0.016
**bio7**	0.755	0.019	0.328	0.056	0.057	0.071	0.23	0.048
**bio9**	0.068	0	0.016	0.039	0.051	0	0.008	0.01
**bio10**	0.94	0.018	0.611	0.061	0.017	0.716	0.306	0.029
**bio11**	0.472	0.001	0.621	0.092	0.169	0.752	0.015	0.022
**bio13**	0.345	0.012	0.06	0.076	0.077	0.753	0.108	0.031
**bio14**	0.013	0.004	0.023	0.007	0.02	0.019	0.008	0.018
**bio15**	0.005	0	0.014	0.003	0.032	0	0	0.013
**bio16**	0.081	0	0.033	0.009	0.088	0.11	0.051	0.024
**bio18**	0.086	0.14	0.085	0.159	0.661	0.039	0.352	0.065
**bio19**	0.009	0.004	0.011	0.043	0.026	0.025	0.015	0.018
**NDVI**	0.051	0.02	0.031	0.034	0.029	0.052	0.042	0.021

Table S11. *Aedes africanus* final model variable importances

	GLM	GBM	GAM	CTA	ANN	FDA	MARS	RF
**bio1**	0.562	0	0.696	0	0.112	0.518	0.062	0.004
**bio2**	0.184	0.005	0.1	0.078	0.165	0	0	0.007
**bio3**	0.418	0.005	0.511	0.138	0.01	0.041	0	0.027
**bio4**	0.999	0.423	0.324	0.717	0.884	0.269	0.548	0.041
**bio7**	0.41	0.005	0.311	0.101	0.349	0	0.018	0.017
**bio8**	0	0.013	0.564	0.229	0.451	0.014	0.361	0.012
**bio9**	0.545	0.002	0.582	0	0.392	0	0.396	0.007
**bio10**	0.69	0.003	0.584	0.141	0.132	0	0.219	0.009
**bio11**	0	0.01	0.571	0.276	0.355	0.972	0	0.01
**bio13**	0.688	0.025	0.747	0.18	0.912	0.871	0.445	0.024
**bio14**	0.301	0.002	0.265	0.013	0.216	0.071	0	0.004
**bio15**	0.391	0.001	0.396	0.038	0.293	0	0	0.005
**bio16**	0.136	0.006	0.398	0.033	0.433	0.356	0.189	0.017
**bio18**	0.162	0.01	0.331	0.021	0.226	0.069	0.069	0.009
**bio19**	0.11	0.127	0.517	0.246	0.541	0.733	0.421	0.047
**NDVI**	0.239	0.065	0.297	0	0.291	0.176	0.21	0.028

Table S12. *Aedes albopictus* final model variable importances

	GLM	GBM	GAM	CTA	FDA	MARS	RF	MAXENT
**bio1**	0.35	0	0.357	0	0.196	0.198	0.01	0.143
**bio2**	0.058	0.032	0.001	0.233	0.003	0.018	0.105	0.094
**bio3**	0.297	0.003	0.069	0.055	0.103	0.189	0.027	0.066
**bio4**	0.416	0	0.312	0	0.025	0.141	0.023	0.005
**bio5**	0.569	0	0.41	0	0.286	0.311	0.026	0.019
**bio6**	1	0	0.45	0	0.041	0.355	0.023	0.001
**bio7**	0.29	0.006	0.181	0	0	0	0.083	0.003
**bio10**	0.761	0.011	0.61	0.01	0.646	0.36	0.012	0.122
**bio11**	0.5	0	0.243	0.007	0.313	0.281	0.013	0
**bio12**	0.046	0.002	0.017	0	0	0	0.014	0.007
**bio13**	0.226	0.001	0.055	0	0	0	0.022	0.16
**bio14**	0.017	0.002	0.012	0	0.008	0.023	0.021	0.028
**bio15**	0.003	0.001	0.001	0.009	0	0.009	0.016	0.004
**bio18**	0.105	0.462	0.07	0.643	0.357	0.113	0.154	0.057
**bio19**	0.002	0.005	0.009	0	0.009	0	0.021	0.021
**NDVI**	0.026	0.007	0.079	0.002	0.022	0.015	0.009	0.058

Table S13. Variable importance in supplementary ZIKV+ model.

	GLM	GBM	GAM	CTA	FDA	MARS	RF
**bio1**	0.34	0.001	0.431	0	0.125	0.046	0.008
**bio2**	0	0.006	0.098	0.143	0	0	0.015
**bio3**	0.223	0.003	0.027	0.231	0.307	0.039	0.024
**bio4**	0.456	0.094	0.819	0	0.16	0.684	0.07
**bio6**	0	0.004	0.344	0	0	0	0.011
**bio7**	0.276	0.025	0	0	0.104	0	0.034
**bio8**	0.822	0.123	0.231	0.604	0.91	0.501	0.058
**bio10**	0.636	0.007	0.549	0.198	0	0.59	0.007
**bio11**	0	0.003	0.994	0	0.036	0.406	0.017
**bio13**	0	0.009	0.04	0.272	0.082	0	0.018
**bio15**	0	0.011	0.167	0.017	0.124	0.328	0.017
**bio16**	0	0.011	0.048	0.061	0.013	0.033	0.012
**bio17**	0	0.003	0.107	0	0	0.133	0.012
**bio18**	0.071	0.008	0.1	0.034	0.023	0.163	0.016
**bio19**	0	0.032	0.14	0.173	0.129	0.111	0.026
**NDVI**	0.184	0.058	0.061	0.082	0.109	0.156	0.04
