# Supplementary material for: An Ecological Assessment of the Pandemic Threat of Zika Virus
Source: PLoS Negl Trop Dis. 2016 Aug 26;10(8):e0004968. doi: 10.1371/journal.pntd.0004968 (PMC5001720; doi:10.1371/journal.pntd.0004968)
Supplement: S5 Table — Variable contributions are based on one preliminary run with 20 variables and 10 candidate models. (PDF) [file pntd.0004968.s005.pdf]

Table S13. Variable importance in supplementary ZIKV+ model.

	GLM	GBM	GAM	CTA	FDA	MARS	RF
**bio1**	0.34	0.001	0.431	0	0.125	0.046	0.008
**bio2**	0	0.006	0.098	0.143	0	0	0.015
**bio3**	0.223	0.003	0.027	0.231	0.307	0.039	0.024
**bio4**	0.456	0.094	0.819	0	0.16	0.684	0.07
**bio6**	0	0.004	0.344	0	0	0	0.011
**bio7**	0.276	0.025	0	0	0.104	0	0.034
**bio8**	0.822	0.123	0.231	0.604	0.91	0.501	0.058
**bio10**	0.636	0.007	0.549	0.198	0	0.59	0.007
**bio11**	0	0.003	0.994	0	0.036	0.406	0.017
**bio13**	0	0.009	0.04	0.272	0.082	0	0.018
**bio15**	0	0.011	0.167	0.017	0.124	0.328	0.017
**bio16**	0	0.011	0.048	0.061	0.013	0.033	0.012
**bio17**	0	0.003	0.107	0	0	0.133	0.012
**bio18**	0.071	0.008	0.1	0.034	0.023	0.163	0.016
**bio19**	0	0.032	0.14	0.173	0.129	0.111	0.026
**NDVI**	0.184	0.058	0.061	0.082	0.109	0.156	0.04
