# Supplementary material for: An Ecological Assessment of the Pandemic Threat of Zika Virus
Source: PLoS Negl Trop Dis. 2016 Aug 26;10(8):e0004968. doi: 10.1371/journal.pntd.0004968 (PMC5001720; doi:10.1371/journal.pntd.0004968)
Supplement: S6 Table — Variable contributions are based on one preliminary run with 20 variables and 10 candidate models. (PDF) [file pntd.0004968.s006.pdf]

Table S6. *Aedes albopictus* full variable set preliminary model variable importance

	GLM	GBM	GAM	CTA	ANN	SRE	FDA	MARS	RF	MAXENT
**bio1**	0.289	0	0.216	0.011	0.09	0.248	0.173	0.006	0.01	0.017
**bio2**	0.06	0.017	0.048	0.031	0.031	0.249	0.025	0.018	0.082	0.012
**bio3**	0.279	0.003	0.277	0.13	0	0.193	0.185	0.261	0.016	0.018
**bio4**	0.528	0	0.352	0.016	0.197	0.242	0	0	0.015	0.008
**bio5**	0.131	0	0.875	0.015	0.007	0.305	0.363	0.202	0.012	0.014
**bio6**	0.262	0	1	0	0.334	0.237	0.626	0.136	0.017	0.019
**bio7**	0.742	0.004	0.607	0.104	0.511	0.256	0.071	0.417	0.05	0.009
**bio8**	0.001	0.001	0.047	0	0.03	0.275	0	0	0.008	0.038
**bio9**	0.007	0	0.1	0	0.049	0.249	0	0	0.008	0.038
**bio10**	0.835	0.01	0.581	0	0.004	0.281	0.619	0.338	0.007	0.017
**bio11**	0.164	0	0.292	0	0.012	0.236	0.624	0.129	0.011	0.023
**bio12**	0.041	0.001	0.009	0.057	0.482	0.299	0	0	0.011	0.015
**bio13**	0.207	0.001	0.274	0	0.295	0.276	0	0	0.01	0
**bio14**	0.029	0.001	0.04	0.003	0.016	0.229	0.04	0.069	0.012	0.01
**bio15**	0.007	0	0.02	0.002	0.004	0.138	0	0	0.015	0.203
**bio16**	0	0	0.051	0.006	0.013	0.278	0	0	0.015	0.002
**bio17**	0.019	0.002	0.027	0	0.037	0.243	0.046	0.039	0.006	0.022
**bio18**	0.147	0.474	0.075	0.377	0.315	0.287	0.44	0.122	0.114	0.066
**bio19**	0.003	0.005	0.011	0	0.012	0.213	0.013	0.008	0.013	0.019
**NDVI**	0.027	0.009	0.021	0.024	0.008	0.26	0.026	0.032	0.008	0.006
