# Supplementary material for: An Ecological Assessment of the Pandemic Threat of Zika Virus
Source: PLoS Negl Trop Dis. 2016 Aug 26;10(8):e0004968. doi: 10.1371/journal.pntd.0004968 (PMC5001720; doi:10.1371/journal.pntd.0004968)
Supplement: S7 Table — Bolded models were shown in the final models. Updated Zika model incorporating New World outbreak data included as “ZIKV+”. (PDF) [file pntd.0004968.s007.pdf]

Table S7. AUC of ten models for five species (with reduced variable sets). Bolded models were shown in the final models. Updated Zika model incorporating New World outbreak data included as "ZIKV+".

	GLM	GBM	GAM	CTA	ANN	SRE	FDA	MARS	RF	MAXENT
A.Aeg	**0.975**	**0.980**	**0.981**	**0.977**	**0.957**	0.855	**0.974**	**0.976**	**1.000**	0.930
A.Afr	**0.983**	**0.999**	**1.000**	**0.985**	**0.967**	0.837	**0.959**	**0.979**	**1.000**	0.739
A.Alb*	**0.919**	**0.942**	**0.938**	**0.945**	0.882	0.760	**0.923**	**0.930**	**1.000**	**0.940**
ZIKV	**0.934**	**0.975**	**0.968**	**0.920**	0.773	0.741	**0.934**	**0.938**	**1.000**	0.807
ZIKV+	**0.920**	**0.975**	**0.946**	**0.921**	--	--	**0.927**	**0.936**	**1.000**	--
DENG*	**0.919**	**0.942**	**0.938**	**0.945**	0.882	0.760	**0.923**	**0.930**	**1.000**	**0.940**
