# Supplementary material for: An Ecological Assessment of the Pandemic Threat of Zika Virus
Source: PLoS Negl Trop Dis. 2016 Aug 26;10(8):e0004968. doi: 10.1371/journal.pntd.0004968 (PMC5001720; doi:10.1371/journal.pntd.0004968)
Supplement: S8 Table — The final ensemble model includes seven modeling methods using sixteen variables, each run for 10 iterations. (PDF) [file pntd.0004968.s008.pdf]

Table S8. Zika final model variable importances

	GLM	GBM	GAM	CTA	FDA	MARS	RF
**bio1**	0.608	0.014	0.654	0	0	0	0.026
**bio2**	0.919	0.006	0.738	0.068	0	0	0.019
**bio3**	0.774	0.02	0.481	0.178	0.1	0	0.029
**bio4**	1	0	0.329	0	0	0	0.017
**bio5**	0	0.006	0.708	0	0	0.279	0.015
**bio6**	0.626	0.211	0	0.879	0.46	0.803	0.125
**bio7**	0	0.033	0.563	0	0.268	0.354	0.051
**bio10**	1	0.003	0.792	0	0	0	0.014
**bio11**	0	0.018	1	0	0	0.033	0.047
**bio12**	0	0.022	0.104	0	0.227	0	0.028
**bio13**	0	0.109	0.075	0	0.462	0.873	0.116
**bio15**	0	0.046	0.207	0.323	0.071	0.295	0.026
**bio16**	0	0.01	0	0	0.153	0.506	0.043
**bio17**	0.258	0.008	0.183	0.094	0.208	0.211	0.013
**bio19**	0	0.01	0.02	0.201	0.062	0.326	0.008
**NDVI**	0	0.082	0.266	0	0.095	0.027	0.06
