# Supplementary material for: An Ecological Assessment of the Pandemic Threat of Zika Virus
Source: PLoS Negl Trop Dis. 2016 Aug 26;10(8):e0004968. doi: 10.1371/journal.pntd.0004968 (PMC5001720; doi:10.1371/journal.pntd.0004968)
Supplement: S9 Table — The final ensemble model includes eight modeling methods using sixteen variables, each run for 10 iterations. (PDF) [file pntd.0004968.s009.pdf]

Table S9. Dengue final model variable importances

	GLM	GBM	GAM	CTA	FDA	MARS	RF	MAXENT
**bio1**	0.388	0.001	0.363	0.004	0.248	0	0.017	0
**bio2**	0.004	0.029	0.054	0.097	0	0	0.053	0.021
**bio4**	0.159	0.012	0.241	0.076	0	0.233	0.06	0.031
**bio6**	0.236	0.008	0.213	0	0.266	0	0.045	0.017
**bio7**	0.702	0.036	0.571	0.328	0.639	0.406	0.073	0.043
**bio8**	0.225	0.009	0.083	0.055	0.006	0.235	0.021	0.084
**bio9**	0.272	0	0.063	0.008	0	0.186	0.012	0.029
**bio10**	0.427	0	0.502	0.033	0.508	0.051	0.015	0.071
**bio11**	1	0.109	0.818	0.527	0.85	0.158	0.062	0.487
**bio13**	0.012	0.008	0.138	0.098	0.072	0.104	0.02	0.001
**bio14**	0.032	0.009	0.082	0.147	0.022	0.028	0.034	0.03
**bio15**	0	0.001	0.031	0.028	0	0	0.011	0.03
**bio16**	0	0	0.075	0.005	0	0	0.016	0.007
**bio18**	0.005	0.002	0.004	0.011	0	0	0.025	0.001
**bio19**	0.032	0.026	0.055	0.064	0.069	0.052	0.048	0.074
**NDVI**	0.095	0.042	0.085	0.17	0.081	0.113	0.05	0.052
