# Supplementary material for: An Ecological Assessment of the Pandemic Threat of Zika Virus
Source: PLoS Negl Trop Dis. 2016 Aug 26;10(8):e0004968. doi: 10.1371/journal.pntd.0004968 (PMC5001720; doi:10.1371/journal.pntd.0004968)
Supplement: S10 Table — The final ensemble model includes eight modeling methods using sixteen variables, each run for 10 iterations. (PDF) [file pntd.0004968.s010.pdf]

Table S10. *Aedes aegypti* final model variable importances

	GLM	GBM	GAM	CTA	ANN	FDA	MARS	RF
**bio1**	0.276	0.01	0.462	0.012	0.019	0.212	0.024	0.02
**bio2**	0.13	0.011	0.05	0.234	0.023	0	0	0.083
**bio3**	0.372	0.005	0.148	0.048	0.008	0.102	0.078	0.018
**bio4**	0.82	0.001	0.198	0.026	0.048	0	0	0.02
**bio5**	0.273	0	0.178	0.012	0.064	0.215	0	0.016
**bio7**	0.755	0.019	0.328	0.056	0.057	0.071	0.23	0.048
**bio9**	0.068	0	0.016	0.039	0.051	0	0.008	0.01
**bio10**	0.94	0.018	0.611	0.061	0.017	0.716	0.306	0.029
**bio11**	0.472	0.001	0.621	0.092	0.169	0.752	0.015	0.022
**bio13**	0.345	0.012	0.06	0.076	0.077	0.753	0.108	0.031
**bio14**	0.013	0.004	0.023	0.007	0.02	0.019	0.008	0.018
**bio15**	0.005	0	0.014	0.003	0.032	0	0	0.013
**bio16**	0.081	0	0.033	0.009	0.088	0.11	0.051	0.024
**bio18**	0.086	0.14	0.085	0.159	0.661	0.039	0.352	0.065
**bio19**	0.009	0.004	0.011	0.043	0.026	0.025	0.015	0.018
**NDVI**	0.051	0.02	0.031	0.034	0.029	0.052	0.042	0.021
