# Supplementary material for: An Ecological Assessment of the Pandemic Threat of Zika Virus
Source: PLoS Negl Trop Dis. 2016 Aug 26;10(8):e0004968. doi: 10.1371/journal.pntd.0004968 (PMC5001720; doi:10.1371/journal.pntd.0004968)
Supplement: S11 Table — The final ensemble model includes eight modeling methods using sixteen variables, each run for 10 iterations. (PDF) [file pntd.0004968.s011.pdf]

Table S11. *Aedes africanus* final model variable importances

	GLM	GBM	GAM	CTA	ANN	FDA	MARS	RF
**bio1**	0.562	0	0.696	0	0.112	0.518	0.062	0.004
**bio2**	0.184	0.005	0.1	0.078	0.165	0	0	0.007
**bio3**	0.418	0.005	0.511	0.138	0.01	0.041	0	0.027
**bio4**	0.999	0.423	0.324	0.717	0.884	0.269	0.548	0.041
**bio7**	0.41	0.005	0.311	0.101	0.349	0	0.018	0.017
**bio8**	0	0.013	0.564	0.229	0.451	0.014	0.361	0.012
**bio9**	0.545	0.002	0.582	0	0.392	0	0.396	0.007
**bio10**	0.69	0.003	0.584	0.141	0.132	0	0.219	0.009
**bio11**	0	0.01	0.571	0.276	0.355	0.972	0	0.01
**bio13**	0.688	0.025	0.747	0.18	0.912	0.871	0.445	0.024
**bio14**	0.301	0.002	0.265	0.013	0.216	0.071	0	0.004
**bio15**	0.391	0.001	0.396	0.038	0.293	0	0	0.005
**bio16**	0.136	0.006	0.398	0.033	0.433	0.356	0.189	0.017
**bio18**	0.162	0.01	0.331	0.021	0.226	0.069	0.069	0.009
**bio19**	0.11	0.127	0.517	0.246	0.541	0.733	0.421	0.047
**NDVI**	0.239	0.065	0.297	0	0.291	0.176	0.21	0.028
