# Supplementary material for: An Ecological Assessment of the Pandemic Threat of Zika Virus
Source: PLoS Negl Trop Dis. 2016 Aug 26;10(8):e0004968. doi: 10.1371/journal.pntd.0004968 (PMC5001720; doi:10.1371/journal.pntd.0004968)
Supplement: S12 Table — The final ensemble model includes eight modeling methods using sixteen variables, each run for 10 iterations. (PDF) [file pntd.0004968.s012.pdf]

Table S12. *Aedes albopictus* final model variable importances

	GLM	GBM	GAM	CTA	FDA	MARS	RF	MAXENT
**bio1**	0.35	0	0.357	0	0.196	0.198	0.01	0.143
**bio2**	0.058	0.032	0.001	0.233	0.003	0.018	0.105	0.094
**bio3**	0.297	0.003	0.069	0.055	0.103	0.189	0.027	0.066
**bio4**	0.416	0	0.312	0	0.025	0.141	0.023	0.005
**bio5**	0.569	0	0.41	0	0.286	0.311	0.026	0.019
**bio6**	1	0	0.45	0	0.041	0.355	0.023	0.001
**bio7**	0.29	0.006	0.181	0	0	0	0.083	0.003
**bio10**	0.761	0.011	0.61	0.01	0.646	0.36	0.012	0.122
**bio11**	0.5	0	0.243	0.007	0.313	0.281	0.013	0
**bio12**	0.046	0.002	0.017	0	0	0	0.014	0.007
**bio13**	0.226	0.001	0.055	0	0	0	0.022	0.16
**bio14**	0.017	0.002	0.012	0	0.008	0.023	0.021	0.028
**bio15**	0.003	0.001	0.001	0.009	0	0.009	0.016	0.004
**bio18**	0.105	0.462	0.07	0.643	0.357	0.113	0.154	0.057
**bio19**	0.002	0.005	0.009	0	0.009	0	0.021	0.021
**NDVI**	0.026	0.007	0.079	0.002	0.022	0.015	0.009	0.058
