# Supplementary figures and images for: An Ecological Assessment of the Pandemic Threat of Zika Virus
Source: PLoS Negl Trop Dis. 2016 Aug 26;10(8):e0004968. doi: 10.1371/journal.pntd.0004968 (PMC5001720; doi:10.1371/journal.pntd.0004968)

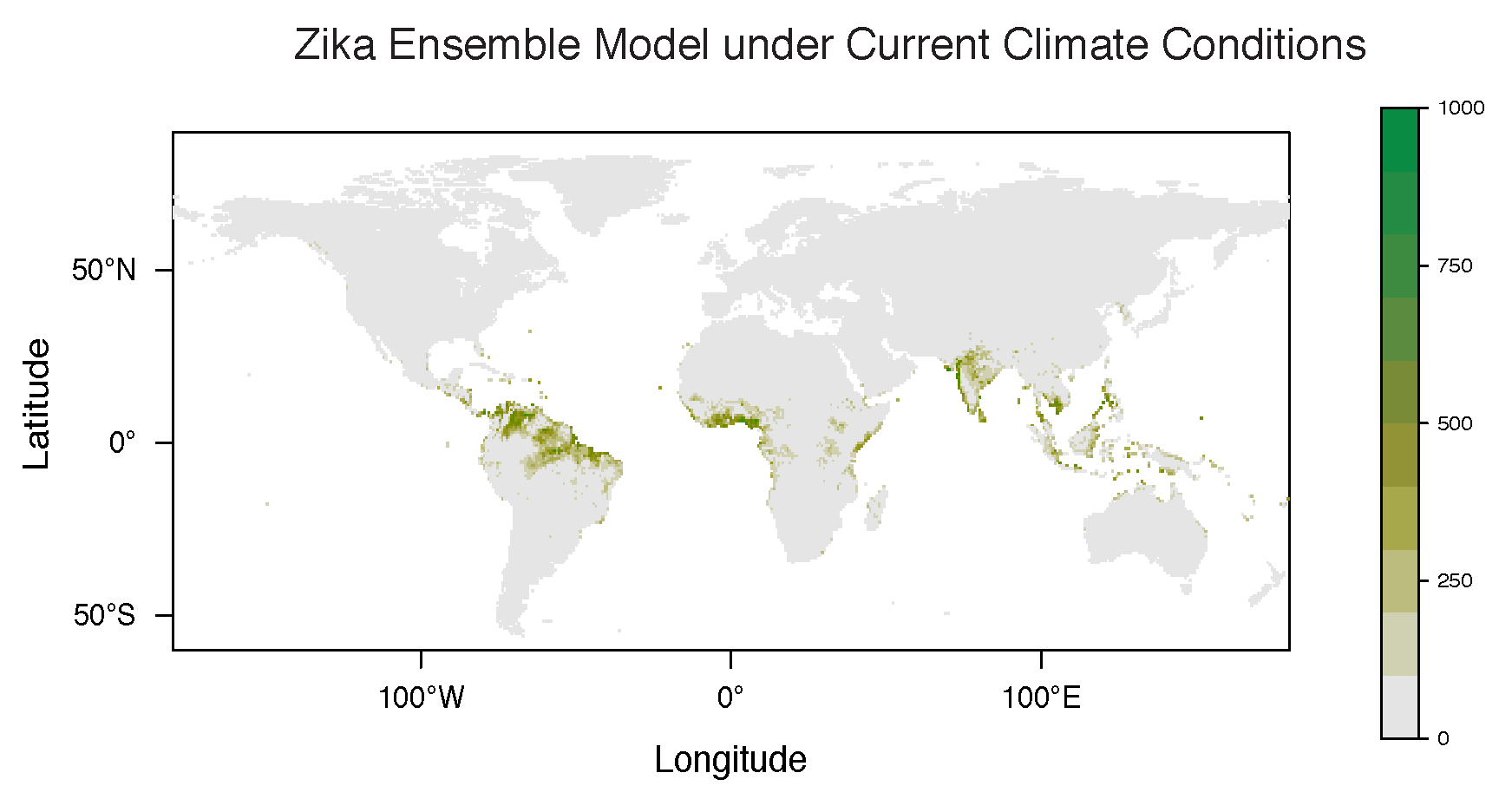

Supplement: S1 Fig — (TIF) [file pntd.0004968.s014.tif]

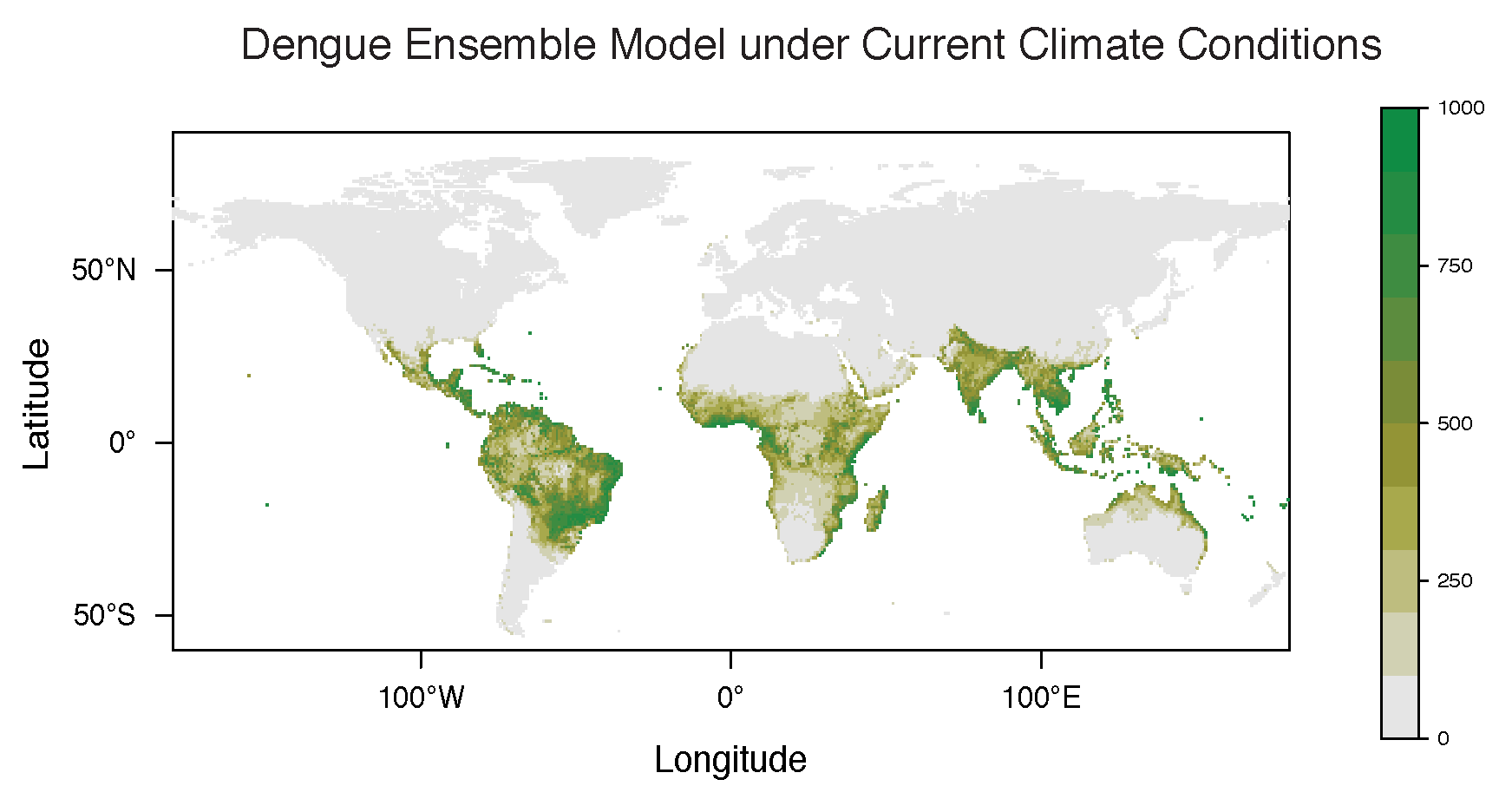

Supplement: S2 Fig — (TIF) [file pntd.0004968.s015.tif]

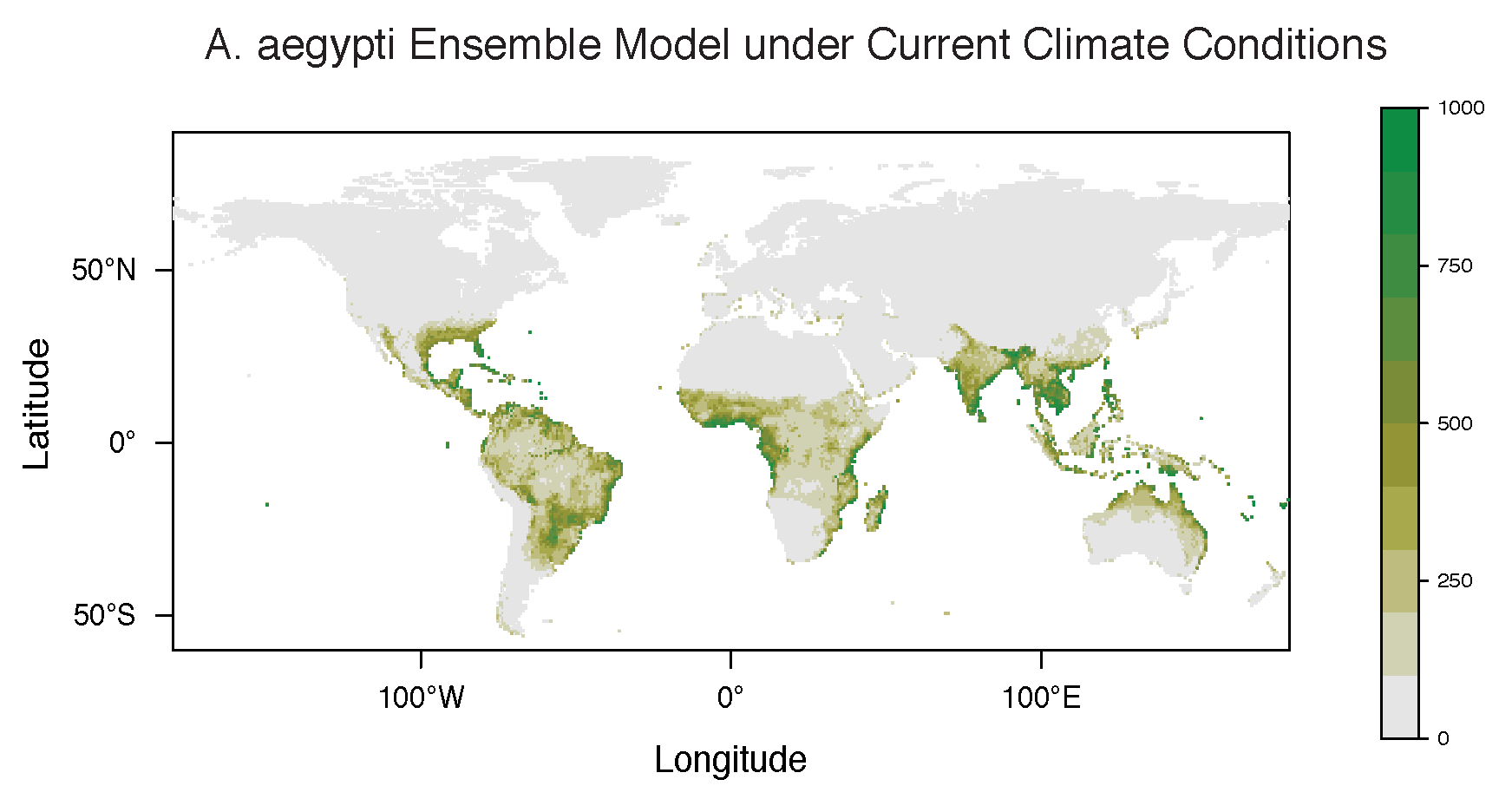

Supplement: S3 Fig — (TIF) [file pntd.0004968.s016.tif]

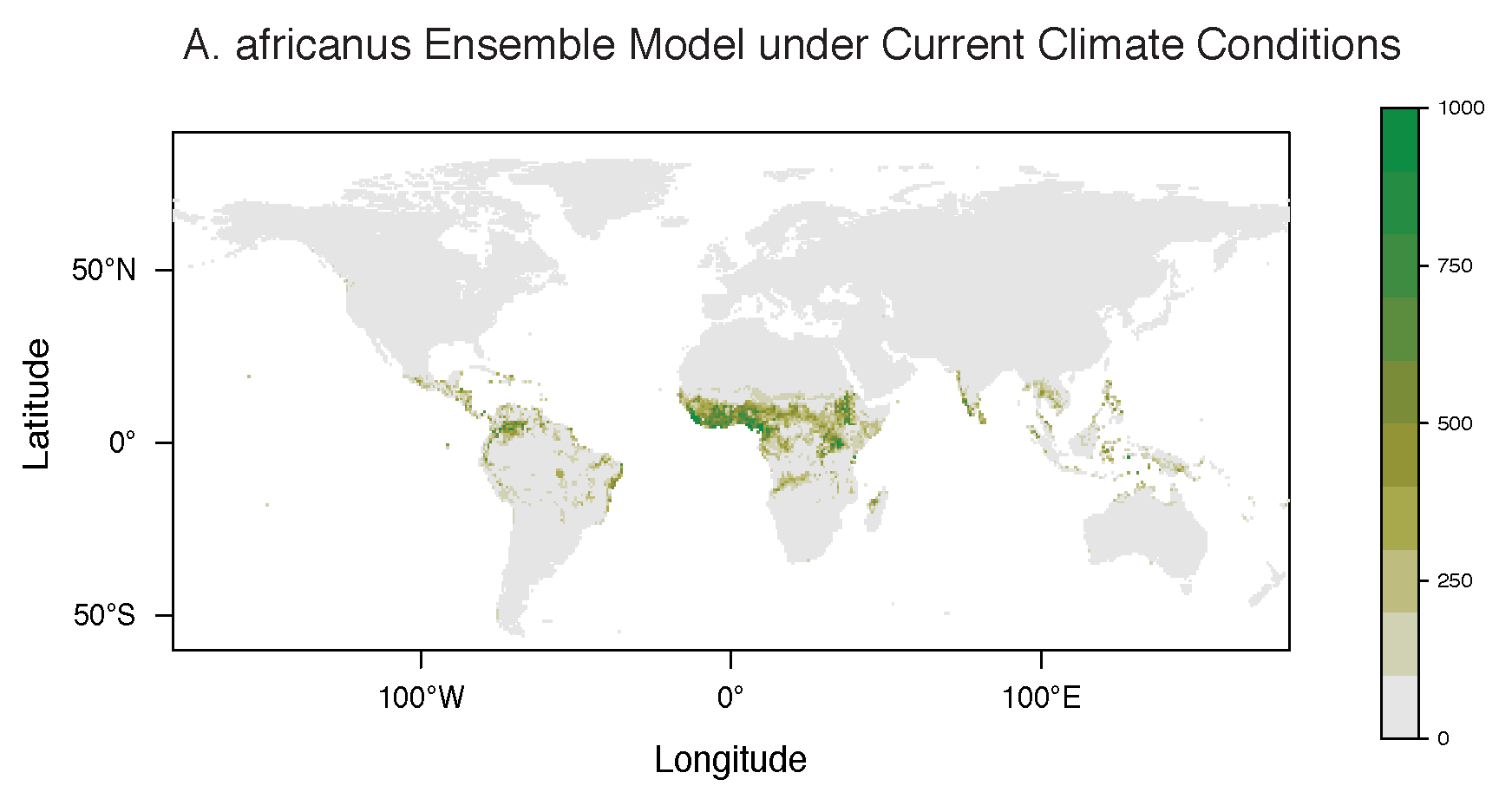

Supplement: S4 Fig — (TIF) [file pntd.0004968.s017.tif]

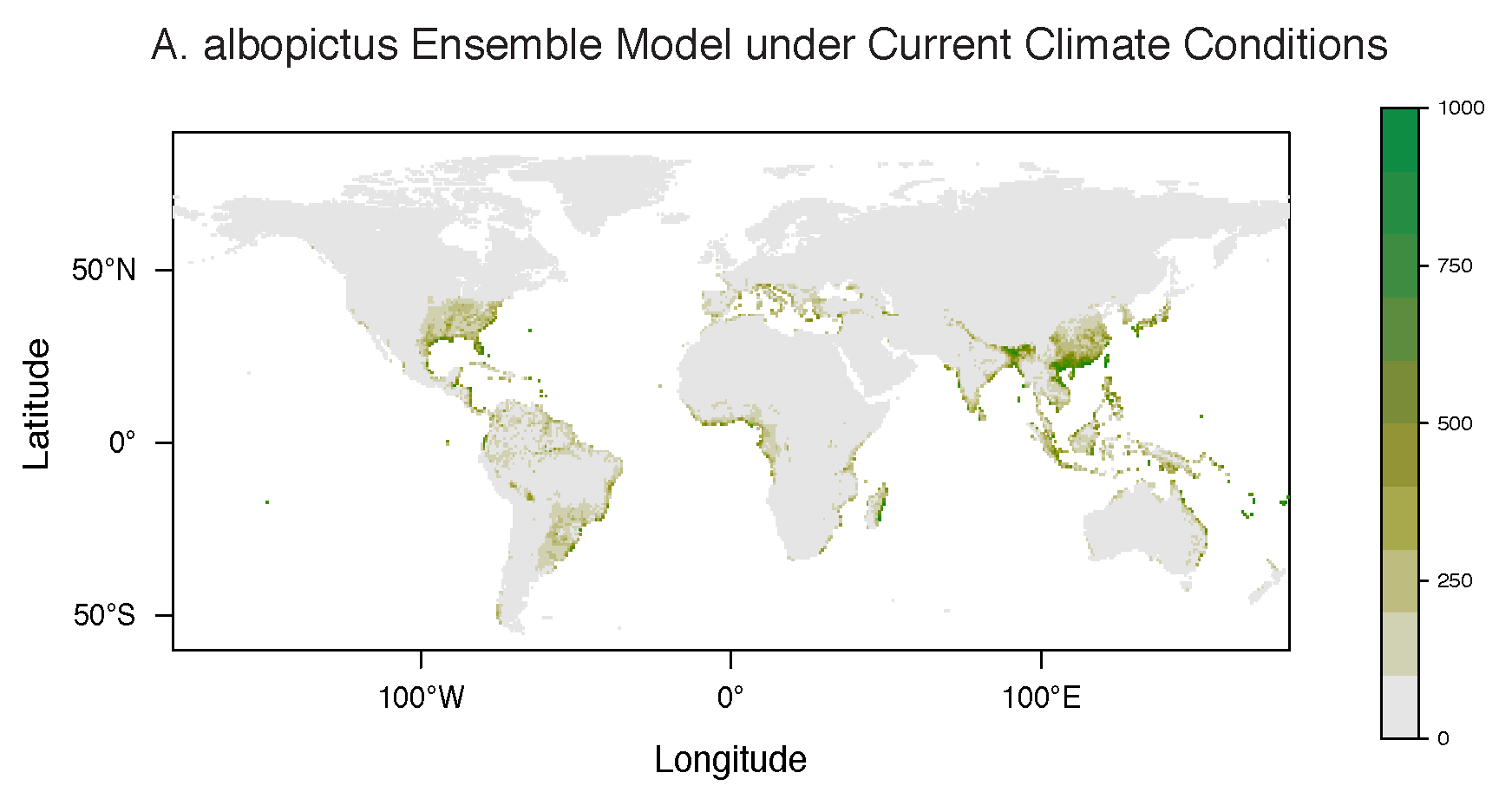

Supplement: S5 Fig — (TIF) [file pntd.0004968.s018.tif]

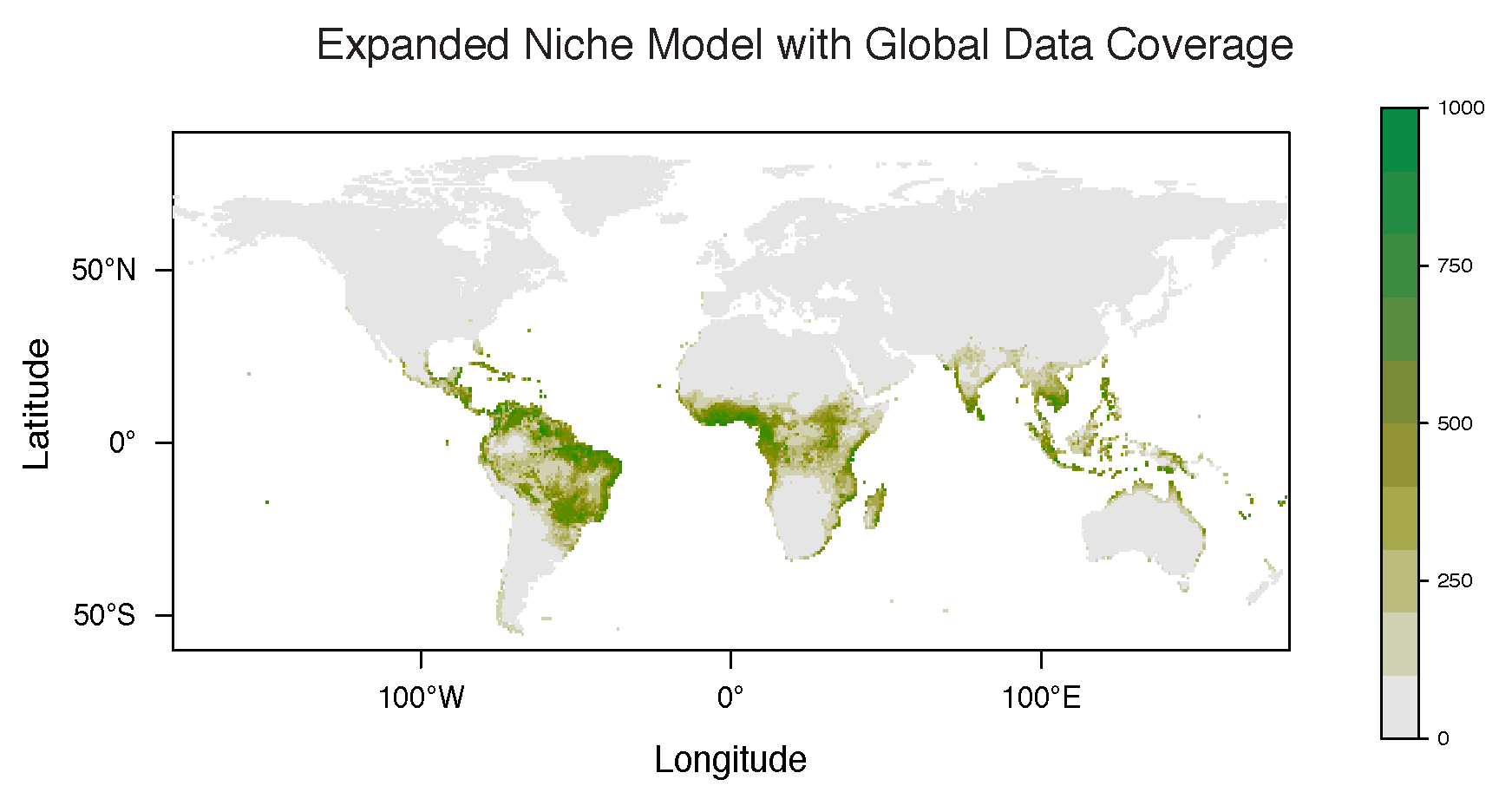

Supplement: S6 Fig — (TIF) [file pntd.0004968.s019.tif]

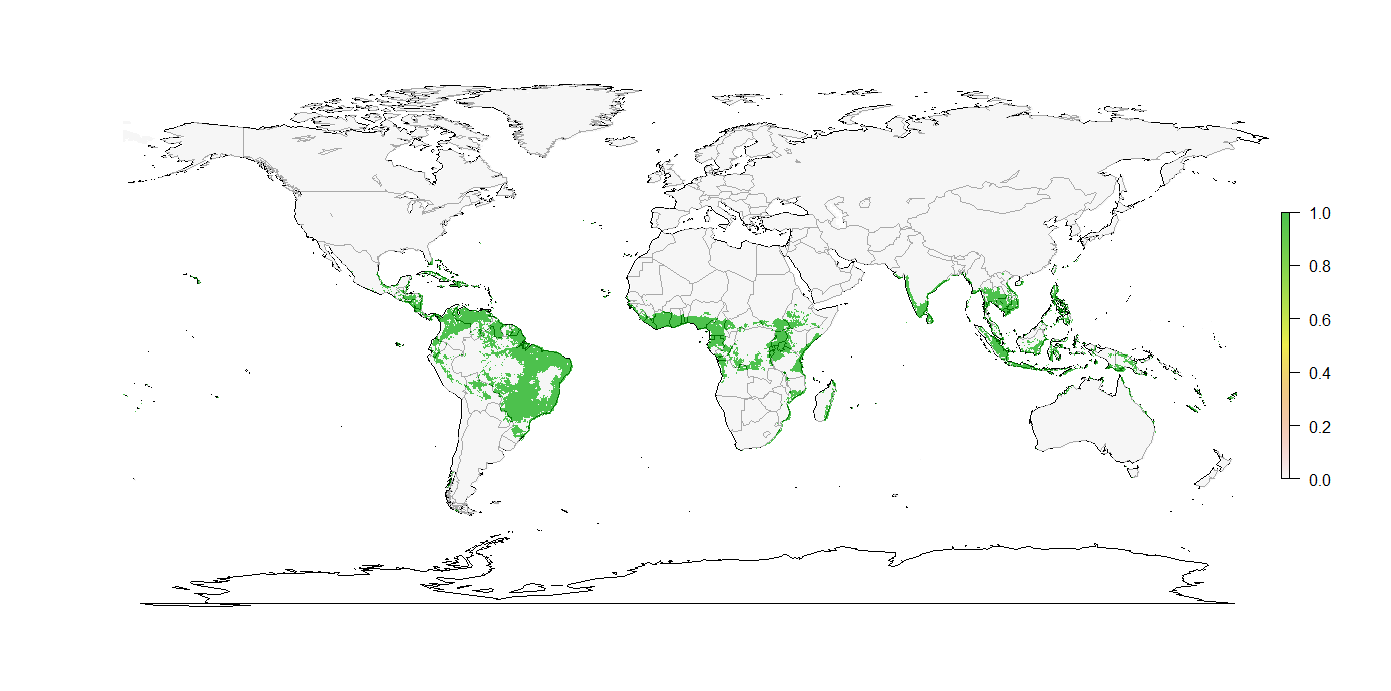

Supplement: S7 Fig — (TIF) [file pntd.0004968.s020.tif]

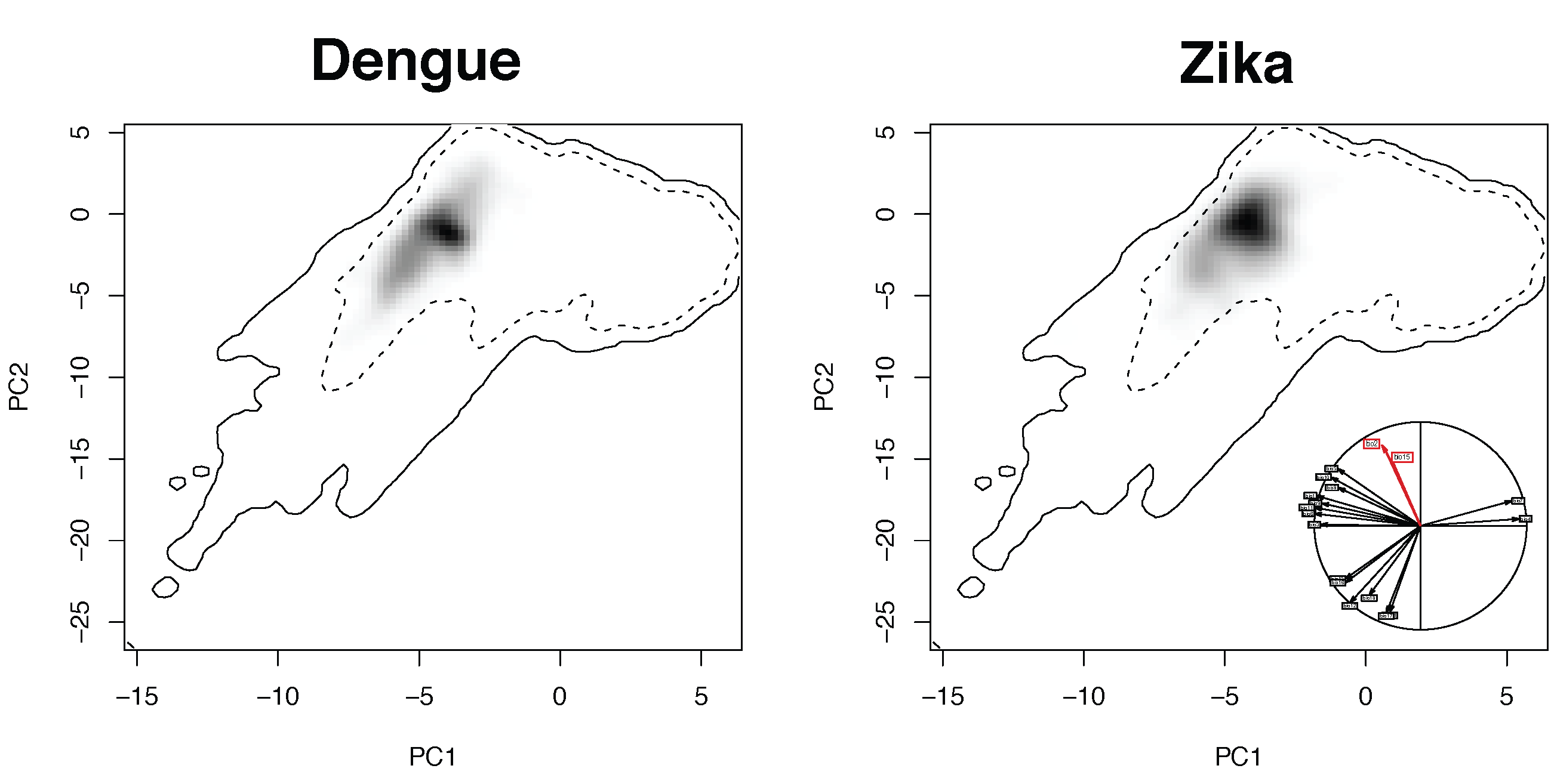

Supplement: S8 Fig — In the equivalency test, we find significant evidence for differences (Schoener’s D = 0.295; p = 0.004). (TIF) [file pntd.0004968.s021.tif]
